# Gene Coexpression Analyses Differentiate Networks Associated with Diverse Cancers Harboring TP53 Missense or Null Mutations

**DOI:** 10.3389/fgene.2016.00137

**Published:** 2016-08-03

**Authors:** Kathleen Oros Klein, Karim Oualkacha, Marie-Hélène Lafond, Sahir Bhatnagar, Patricia N. Tonin, Celia M. T. Greenwood

**Affiliations:** ^1^Lady Davis Research Institute, Jewish General HospitalMontreal, QC, Canada; ^2^Département de Mathématiques, Université du Québec à MontréalMontreal, QC, Canada; ^3^Department of Epidemiology, Biostatistics and Occupational Health, McGill UniversityMontreal, QC, Canada; ^4^Cancer Research Program, The Research Institute of the McGill University Health CentreMontreal, QC, Canada; ^5^Departments of Medicine and Human Genetics, McGill UniversityMontreal, QC, Canada; ^6^Departments of Oncology and Human Genetics, McGill UniversityMontreal, QC, Canada

**Keywords:** TP53, ovarian cancer, breast cancer, lung cancer, melanoma, weighted gene expression network, KIR3DL2, immune pathways

## Abstract

In a variety of solid cancers, missense mutations in the well-established TP53 tumor suppressor gene may lead to the presence of a partially-functioning protein molecule, whereas mutations affecting the protein encoding reading frame, often referred to as null mutations, result in the absence of p53 protein. Both types of mutations have been observed in the same cancer type. As the resulting tumor biology may be quite different between these two groups, we used RNA-sequencing data from The Cancer Genome Atlas (TCGA) from four different cancers with poor prognosis, namely ovarian, breast, lung and skin cancers, to compare the patterns of coexpression of genes in tumors grouped according to their TP53 missense or null mutation status. We used Weighted Gene Coexpression Network analysis (WGCNA) and a new test statistic built on differences between groups in the measures of gene connectivity. For each cancer, our analysis identified a set of genes showing differential coexpression patterns between the TP53 missense- and null mutation-carrying groups that was robust to the choice of the tuning parameter in WGCNA. After comparing these sets of genes across the four cancers, one gene (KIR3DL2) consistently showed differential coexpression patterns between the null and missense groups. KIR3DL2 is known to play an important role in regulating the immune response, which is consistent with our observation that this gene's strongly-correlated partners implicated many immune-related pathways. Examining mutation-type-related changes in correlations between sets of genes may provide new insight into tumor biology.

## Introduction

### The TP53 gene and cancer

It is well-established that mutations of the TP53 gene, which renders loss of function of encoded p53 tumor suppressor protein, is the most frequent somatic genetic anomaly observed in human cancers (Muller and Vousden, 2013). This has recently been verified by the application of whole exome sequencing technology to identify the mutational spectrum of a variety of cancers as exemplified by The Cancer Genome Atlas (TCGA) Research Network (http://cancergenome.nih.gov). The TP53 mutation frequency varies across cancer types, with high-grade serous ovarian carcinomas exhibiting the highest frequency at 96% (Bell et al., 2011; Cerami et al., 2012; Gao et al., 2013), suggesting that TP53 mutations are critical drivers for the development of this subtype of ovarian cancer (Ahmed et al., 2010).

TP53 encodes a DNA-binding transcription factor that induces cell growth arrest, senescence and cell death by apoptosis upon cellular stress (Freed-Pastor and Prives, 2012; Muller and Vousden, 2013). This process serves to eliminate severely damaged or emerging tumor cells. As a consequence of acquiring somatic TP53 mutations, tumor cells are thus able to evade apoptosis and senescence, and progress to more malignant phenotypes. However, unlike other established tumor suppressor genes, such as RB1, which are most commonly inactivated by frame-shift or nonsense mutations, TP53 often encodes mutant proteins as a consequence of missense mutations with a single base-pair change in the coding sequence. The mutant p53 protein, having a prolonged half-life relative to the normal isoform, is able to accumulate in tumor cells, and thus is readily detectable by immunohistochemistry. The majority of missense variants are a consequence of mutations occurring in exons 4-9 which would affect the DNA-binding domain of the protein. The functional consequences of mutant p53 protein are the subject of intense research (reviewed in Brosh and Rotter, 2009; Freed-Pastor and Prives, 2012; Muller and Vousden, 2013), which has shown, using various cell line models, that mutant p53 protein can bind and inactivate p53-related proteins. Moreover, it has been shown that some mutant p53 proteins have acquired new oncogenic functions through interactions with other transcription factors. The consequences of a missense mutation are in stark contrast to frame-shift, nonsense and splice-site mutations in TP53 that have also been observed in a significant fraction of human tumors. This latter set of variants is collectively referred to as p53-null mutations, as they affect the reading frame, resulting in the absence of an encoded protein. Both types of mutations have been detected in the same cancer type, where missense mutations are more commonly observed than the other types. In high-grade serous ovarian carcinomas, we and others have shown that among TP53 mutation-positive tumor samples, between 60 and 70% harbor TP53 missense mutations, and the remaining 30–40% harbor p53-null mutations (Ahmed et al., 2010; Cancer Genome Atlas Research, 2011; Wojnarowicz et al., 2012).

### Links between TP53 mutation subtypes and clinical outcomes

Past attempts to correlate TP53 mutation status with various clinical parameters, such as overall outcome or response to therapy, has often resulted in conflicting results. This is in large part due to an overly superficial examination of the mutations, i.e., by limiting mutation analyses to those exons that encode the DNA binding domain, or by inferring mutation status by immunohistochemistry, which detects tumor cells harboring stable mutant p53 protein but does not readily distinguish those harboring p53 null variants from wild-type variants. However, evidence is emerging that the type of TP53 mutation is associated with differences in clinical outcome. Older studies also did not consider the biological consequences resulting from the nature of the somatic TP53 mutation, which would distinguish cancer cases with TP53 missense mutations from those with null mutations. For example, our group has shown that the subgroup of high-grade serous ovarian carcinomas expressing mutant p53 protein exhibited significantly prolonged overall disease-free survival as compared with carcinomas harboring p53 null mutations (Wojnarowicz et al., 2012). Our results were consistent with earlier reports associating high-grade serous ovarian carcinomas harboring p53 null mutations with poorer overall outcome (Kobel et al., 2008) or distant metastasis (Sood et al., 1999). In contrast, a recent study using a subset of the gene expression data from TCGA set for high grade serous ovarian cancers, has provided some evidence that patients with gain-of function p53 mutant proteins are characterized by a greater likelihood of platinum treatment resistance and distance metastasis (Kang et al., 2013). Thus TP53 mutations may exert different effects on tumor progression and possible chemoresistance in the development of ovarian cancer. This notion is supported in part by our observation of significant genomic copy number differences of specific chromosomal regions in a comparative analysis of high grade serous ovarian cancer harboring p53 missense with those harboring p53 null mutations (Wojnarowicz et al., 2012).

### Network-based analyses of gene expression

When many genes in a co-regulated set are altered subtly but in a coordinated fashion, tests of differential expression performed separately on each gene may have low statistical power to detect differences of interest. In contrast, analyses that explicitly examine evidence for coexpression might provide a clearer picture of how cell regulation has been affected.

Starting from a point of view inspired by knowledge of metabolism, Ravasz et al. (2002) used network theory arguments to develop a new metric of similarity for metabolic networks. They showed that such networks were neither completely modular, nor did they follow a completely scale-free network, but that they demonstrated hierarchical network modularity. Through this work, they proposed use of the Topological Overlap Matrix (TOM), which measures how strongly two nodes are connected to the same set of neighbor nodes. The TOM matrix concept was then applied to gene expression data (Zhang and Horvath, 2005), who showed that an appropriately transformed TOM matrix, built from gene expression correlations, could demonstrate scale-free network properties. These authors developed a clustering method, Weighted Gene Coexpression Network Analysis (WGCNA), for identification of gene modules showing high coexpression, i.e., strong network connectivity.

**Figure 1:**
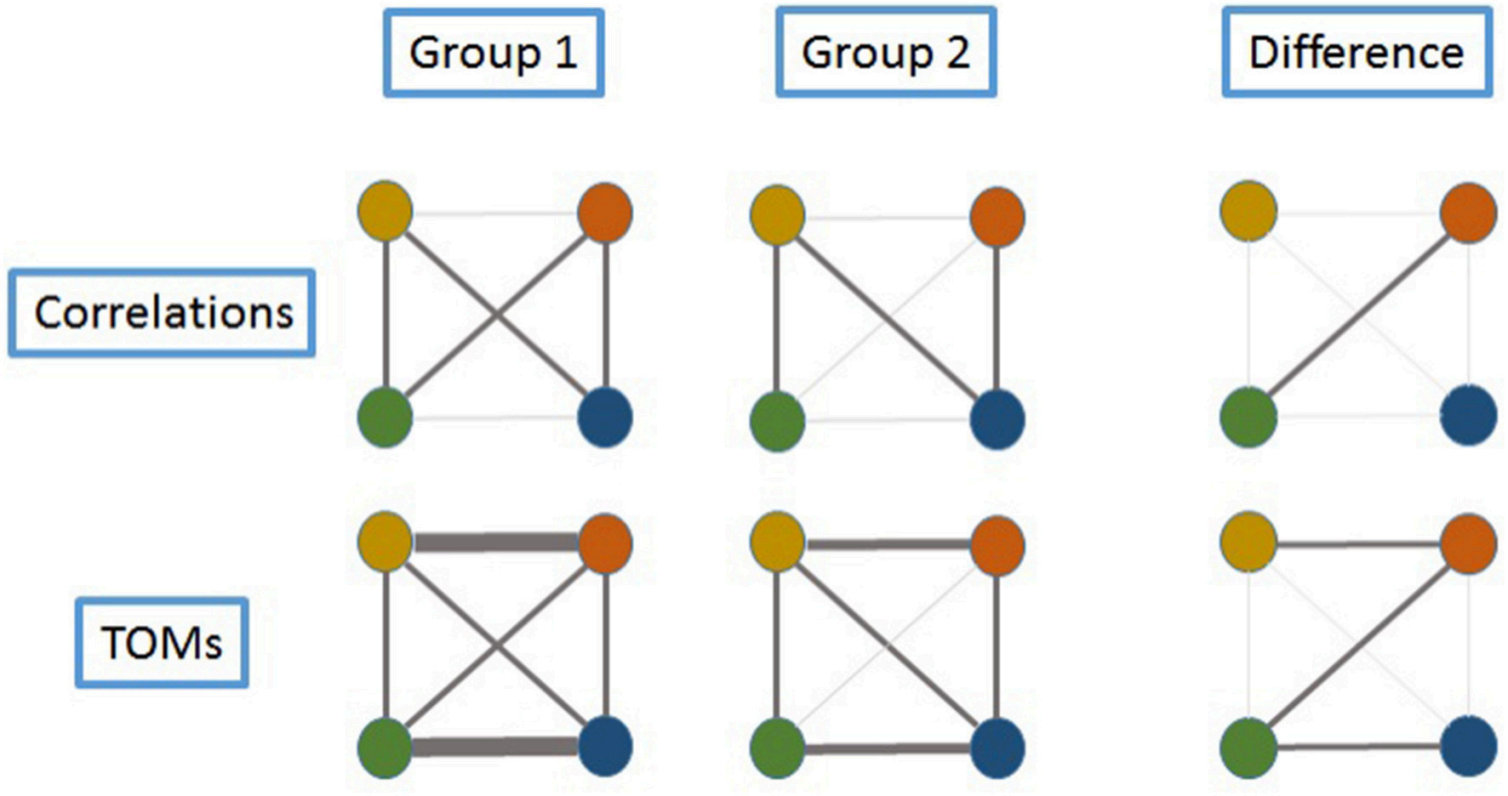
**Schema exemplifying the advantage of using TOMs to identify the relationships in the expression of various genes**. Four genes are represented by colored circles. Assume gene expression is measured in samples from two groups, and the strength of the relationships between genes is indicated by the width of the connecting lines. In Group 1, there is very little correlation between the (blue, green) and (yellow, orange) pairs, as can be seen in the top row of diagrams. Since TOMs also capture indirect relationships between genes, the lower row of TOM-connectivity shows that in fact these theses pairs can be considered as strongly connected. For example, between the green and blue genes, connection can be established via green-yellow-blue and green-orange-blue. In Group 2, correlation between (green, orange) is reduced. There is still a TOM connection between green and blue, via yellow, but it is reduced in strength when compared to Group 1. An examination of differences (last column) would highlight only the (green, orange) pair when using correlations, whereas by using TOMs, a larger set of differentially-connected genes is captured.

To give a heuristic description of this idea, examine Figure 1 where we assume gene expression is measured at four genes, represented by colored circles. In the top left diagram, pairwise correlations between gene expression levels are represented by the widths of the lines, and it can be seen that there is very little pairwise correlation between the (blue, green) and (yellow, orange) pairs. However, indirect relationships between these pairs of genes exist; for example, between the green and blue genes, a relationship can be established via green-yellow-blue and green-orange-blue. Hence, the concept behind the TOM is represented schematically in the bottom left diagram, where the strong blue-green relationship is formed by summing the strengths of the two indirect paths.

In order to explore how co-regulated sets differ between groups, several methods have been proposed. Lai et al. (2004) constructed an extended *F* test that includes consideration of the correlations between the members of the sets, however this is built on a multivariate normal model and may be sensitive to distributional assumptions. Then in 2005, a correlation-based score was developed that highlights genes where correlations are particularly different between groups(Dettling et al., 2005). In parallel, in a series of papers by Yakovlev and colleagues (Szabo et al., 2003; Xiao et al., 2004), a non-parametric measure of variability in correlations was developed; this approach includes clustering to find subsets of genes showing variability. Their measure is built on Fisher-transformed correlations, considered both within and between groups, and was extended in 2009 (Hu R. et al., 2009) to allow comparisons between two groups. In contrast to these methods that are based on correlations, in 2010, a redefined TOM matrix was constructed from differences in correlations between groups (Tesson et al., 2010), i.e., weighted, signed differences in gene expression correlations (between two groups) were used to construct a new TOM matrix that would allow clustering of genes based on notable inter-group network alterations.

### TP53 mutation types and coexpression in four common cancers

To further explore the biological consequences of the TP53 mutation type, we began by examining the gene expression profiles of high-grade serous ovarian carcinomas available from TCGA. We focused our analysis only on samples with TP53 mutations and compared profiles parsed according to the major consequences of TP53 mutation. Although we did test for differential expression between the TP53 mutation types, we felt that such an analysis did not tell a complete story about the patterns of gene expression. Therefore, we hypothesized that differences in gene regulation in the context of TP53 missense or null tumors might be better captured by an alternative analysis based on changes in gene coexpression patterns. We built TOM matrices using the WGCNA method and software (Zhang and Horvath, 2005; Langfelder and Horvath, 2008), and examined their differences using a novel measure. Hence, we identified an interesting set of differentially coexpressed genes. To determine if our observations were unique to this cancer type, we repeated our analyses with three very different types of solid cancers: breast (triple negative), lung (adenocarcinomas), and skin cutaneous melanomas. These cancer types were selected because they exhibit a high frequency of somatic TP53 mutations and RNA-sequencing data were available from TCGA. Although the etiology of high-grade serous ovarian cancer is unknown, breast cancers that are negative for biomarkers that detect the expression of receptors for estrogen (ER), progesterone (PR), and the hormone epidermal growth factor receptor 2 (HER) (i.e., triple negative breast cancers) exhibit molecular genetic features overlapping this subtype of ovarian cancer (Cancer Genome Atlas, 2012; http://www.cancer.gov/types). In contrast, both lung (non small cell) adenocarcinomas and skin cutaneous melanomas are largely caused by environmental factors, i.e., smoking and ultraviolet radiation, respectively (http://www.cancer.gov/types). Finally, our approach suggests a cross-cancer role for one gene in the immune system (KIR3DL2), with TP53 mutation-type- and cancer-type-dependent co-regulation partners.

## Materials and methods

### Gene expression measures

Gene summary expression files from RSEM-normalized(Li and Dewey, 2011) (https://wiki.nci.nih.gov/display/TCGA/RNASeq+Version+2) RNA sequencing experiments, obtained with the Illumina HiSeq platform, were downloaded from The Cancer Genome Atlas (TCGA) portal (http://cancergenome.nih.gov) for each of our four cancers types of interest: high grade serous ovarian cancer (OV), triple negative breast cancer (BRCA), skin cutaneous melanoma (SKCM), and lung (non small cell) cell adenocarcinoma (LUAD). Ovarian cancer data was obtained December 10, 2012, and data for the other cancers in January 28, 2015 (Supplemental Table S1). Table 1 shows the number of samples for which RNA-sequencing data were available for each cancer type. The somatic TP53 mutation status has been reported for most of the cancer types, and this information was obtained from the Open-Access Validated Somatic Mutation Data available from the TCGA portal. For the breast cancer samples, we focused on the triple negative subgroup where the prognosis is poor, as inferred from the immunohistochemistry results for biomarkers that detect the expression of the receptors ER, PR and HER, available from the clinical data.

**Table 1:** **Number of samples, characteristics of the samples, and results of some analyses, by cancer type**.

**Cancer type**	**High grade serous Ovarian carcinomas**	Triple negative breast cancers^**^	Skin cutaneous melanoma^*^	**Lung adenocarcinoma**
**TCGA label**	**OV**	**BRCA**	**SKCM**	**LUAD**
Number of samples with RNA-sequencing data	536	106	470	516
Number of samples with TP53 mutations	171	80	59	260
Number Male/Female	0/171	0/80	38/21	122/138
Number of samples with missense/null mutations	106/65	37/43	33/26	154/106
Number of genes analyzed after removing quartile with lowest coefficient of variation	14,939	14,788	14,683	14,967
Number of genes with *P* < 0.05 for *Sg*, showing evidence of differential coexpression, for different soft threshold parameter (SP) choices	SP3: 327	SP4: 911	SP3: 1385	SP4: 316
	SP4: 333	SP5: 910	SP4: 1282	SP5: 371
	SP5: 343	SP6: 901	SP5: 1296	SP6: 407
	SP6: 378	SP7: 930	SP6: 1345	SP7: 435
		SP8: 1013		

* 51 of the 59 SKCM samples were metastatic melanoma;

** *Negative for ER, PR, and HER*.

### Classifying TP53 missense and null mutations

The mutations were classified into two groups, either null or missense mutations, based on a review of the somatic mutations reported for each cancer. The p53 null group contained frame-shift, nonsense and splice-site mutations, as a consequence of intragenic nucleotide insertions or deletions and/or single base-pair substitutions, which are expected to affect protein encoding reading frames and have been associated with unstable transcripts and lack of protein (Wojnarowicz et al., 2012). Most of the remaining mutations were expected to be p53-expressing missense isoforms which would largely exhibit stable gene and protein expression (Wojnarowicz et al., 2012), and these were assigned to our p53-missense group. Only samples with known TP53 mutations were used in our analyses (Table 1).

### Identifying changes in coexpression of genes

For each dataset we removed genes with very low variability by filtering using the coefficient of variation (standard deviation divided by the mean); genes with coefficient of variation below the first quartile were removed, and we applied a log2(x+1) transformation to the expression data. Then, for each cancer type, we used WGCNA software to calculate matrices of coexpression of size *GxG*, for *G* genes, separately for the missense and null groups, by calculating the unsigned topological overlap matrices (TOM) using the WGCNA package available from http://bioconductor.org. The overlap measure in a TOM matrix of expression, *T*_*gj*_ between genes *g* and *j*, is defined as

(1) $$\begin{array}{l} T_{gj}=\;\frac{\sum_{l=1}^{G}a_{gl}a_{lj}+a_{gj}\;}{\text{min}\left(\sum_{l=1}^{G}a_{gl}\sum_{l=1}^{G}a_{lj}\right)+1-a_{gj}} \end{array}$$

where $a_{gj}={\left|r_{gj}\right|}^{\beta }$ is a power transformation of the correlation, *r*_*gj*_, between the expression levels of genes *g* and *j*. The numerator measures the network strength between genes *g* and *j* by summing the product of the strengths of the connections to all potential partners. The exponent, β, is referred to as the soft threshold (Zhang and Horvath, 2005), and if β > 1, this has the effect of down-weighting small correlations relative to large ones, and hence giving most importance to the strongest pairwise correlations.

Let *T*_*gjm*_ and *T*_*gjn*_ denote the elements of these TOM matrices for null- (*n*) and missense- (*m*) carrying tumors, for a pair of genes (*g, j*); these are calculated after choosing a value for the soft threshold, β, and this will be discussed further below. We have defined a statistic for gene *g, g* = 1, …*G*, that captures the difference in the coexpression patterns between null and missense tumors by:

(2) $$\begin{array}{l} S_{g}=\sum_{j=1}^{G}|T_{gjm}-T_{gjn}|\; \end{array}$$

The motivation for this statistic is represented schematically in Figure 1. Since the distribution of this statistic is unknown, and will depend partially on the level of expression of gene *g* as well as its variability and correlation, we estimated statistical significance, separately for each cancer type, by performing 1000 permutations of the null and missense labels across tumors within the same cancer type, and recalculating the *S*_*g*_ statistics for each gene. Genes with statistical evidence for differential coexpression were defined as those with empirical *p* < 0.05.

### The impact of the soft threshold parameter

During the construction of the TOM matrices, that are built on the transformed correlation measures, $a_{gj}={\left|r_{gj}\right|}^{\beta }$, the WGCNA authors recommend that the soft threshold, β, should be chosen so that the TOM matrix resembles a scale-free network, i.e., containing a small number of hub genes that are connected to many others, and where the majority of the genes display only sparse connections. Since our statistics, *S*_*g*_, are calculated from two TOM matrices, the best choice of the soft threshold might differ between the TOM matrices of the null and missense groups, however it would not make sense to compare overlap measures *T*_*gjm*_ and *T*_*gjn*_ if they were calculated with different soft thresholds. We performed some simulations to study the behavior of the *S*_*g*_ statistics with a range of soft threshold choices—albeit the same threshold for the two matrices within one simulation (see Supplemental Methods and Supplemental Figure S1), and found substantially different scales and skewness in the distributions of *S*_*g*_ with different soft thresholds.

Therefore, when calculating our *S*_*g*_ statistics on the tumor sample data, we were careful to use the same value of the tuning parameter, β, for both mutation types. Given that different optimal values for β might apply to the null- and missense-carrying groups, we constructed both groups' TOM matrices with the same β, and repeated this construction for a range of values. The lower bound for β followed the suggestion by the WGCNA authors, based on the function “pickSoftThreshold” (the minimum suggested value from separate analyses of null- and missense-carrying tumors). As the WGCNA tutorial (https://labs.genetics.ucla.edu/horvath/CoexpressionNetwork/Rpackages/WGCNA/Tutorials/FemaleLiver-02-networkConstr-auto.pdf) suggests, the upper bound for β was lowest value for which the curve of the scale-free topology fit indices appears to reach an asymptote; here we chose the maximum of the two upper bounds between null and missense tumors. Our choice of upper bound ensured that both networks maintained a reasonably high mean number of connections. If the soft threshold were set too high, then the network would demonstrate very few connections (Zhang and Horvath, 2005). We also defined an “optimal” soft threshold when both the coexpression overlap matrices for missense and null tumors reached at least an *R*^2^ of 0.90 when assessing fit to expectation derived from a scale-free topology (see also Supplemental Methods).

We, therefore, performed analyses of each tumor type with a range of values for the soft threshold. In order to assess significance, we permuted the mutation type across tumors, and repeated the calculations of the two TOM matrices and the statistic *S*_*g*_ for the same range of soft thresholds, and for each cancer type. Statistical significance was first estimated by comparing *S*_*g*_ to the empirical distribution from 1000 permutations using the same soft threshold value and the same cancer type, and then by looking for consistency of significance across soft threshold values.

### Gene set enrichment analyses of selected genes

Genes that were identified as differentially connected or coexpressed, for all soft thresholds and across all cancer types, were selected for gene set enrichment analysis. We performed gene set enrichment analysis using both Kyoto Encyclopedia of Genes and Genomes (KEGG) pathways and Gene Ontology (GO) terms for biological processes for each cancer type using the Bioconductor “clusterProfiler” package (Yu et al., 2012).

## Results

### Overview of sample sets for data analyses and mutation types

**Figure 2:**
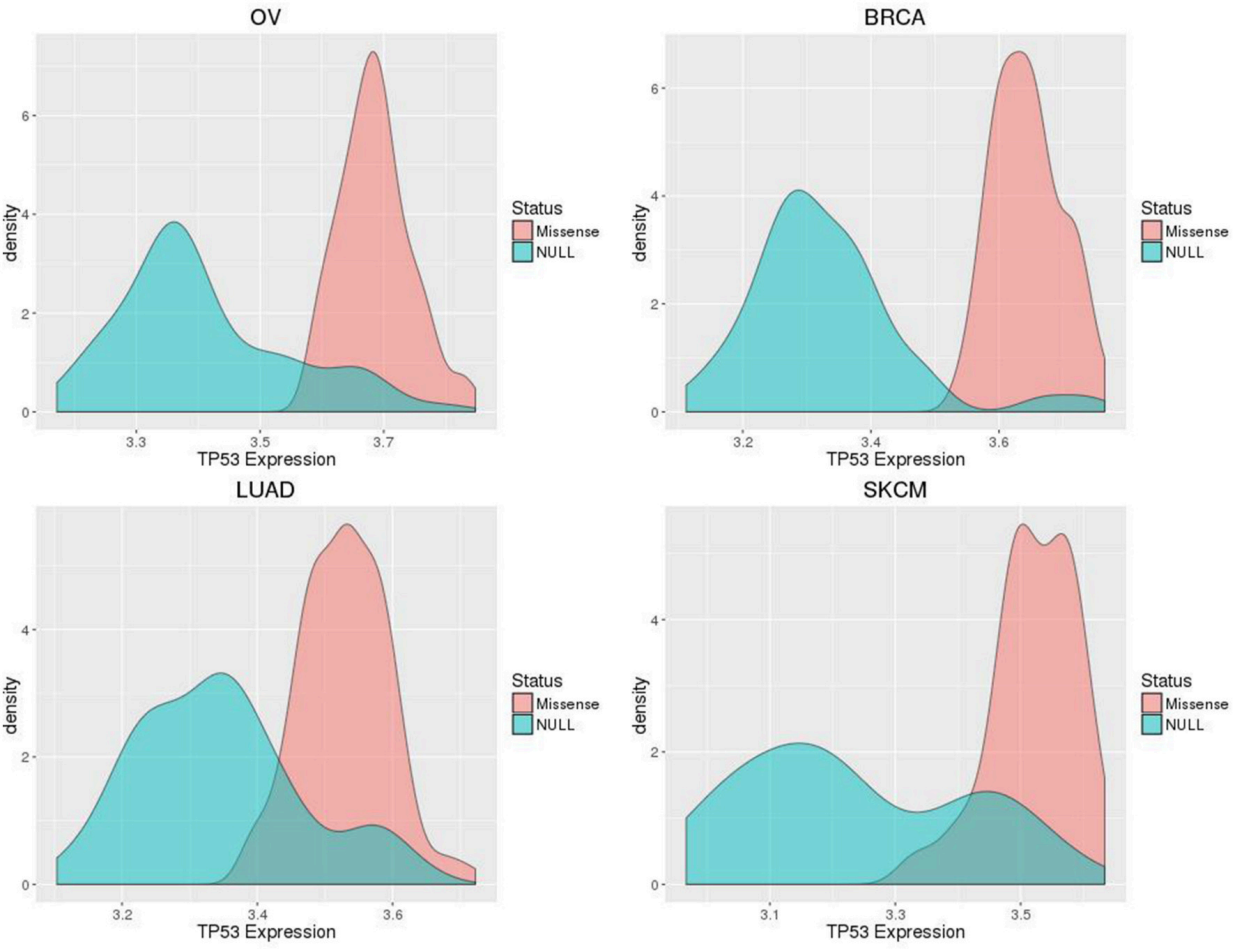
**Smoothed density plots display distributions of TP53 expression separated by assigned mutation classification (p53 missense or null), for ovarian (OV), breast (BRCA), and lung (LUAD) cancers, and skin melanomas (SKCM)**.

The number of samples harboring somatic TP53 mutations available for our study ranged from 59 for SKCM to 260 for LUAD (Table 1). OV had the largest number of samples where missense variants were found, at 62% of all TP53 mutation-positive cases. However, the distribution of each of the two categories of TP53 mutations for each type of cancer was similar, ranging from 46 to 62% for the number of cases harboring missense variants (Table 1). Although immunohistochemistry staining for p53 protein would further distinguish null and missense mutated samples, in the absence of such data, our method of classification shows good separation of gene expression distributions for each cancer type (Figure 2).

### Analyses of each cancer type parsed according to TP53 mutation type

**Figure 3:**
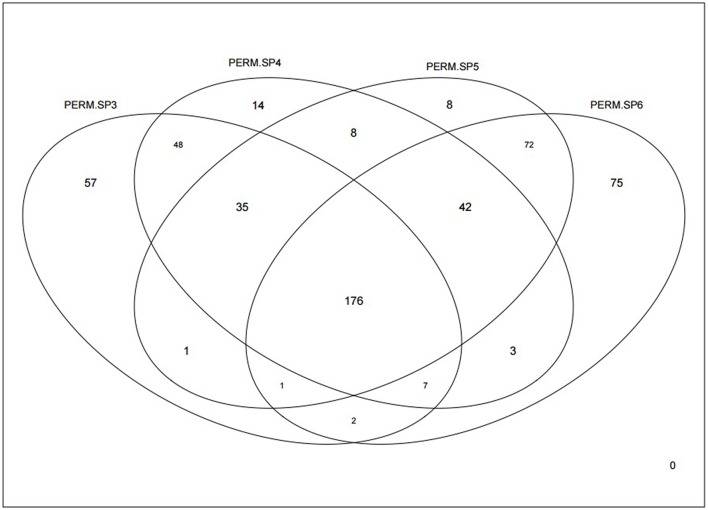
**Venn diagram showing how many genes were identified as significantly differentially coexpressed between the p53 missense and null mutations groups of ovarian cancers, based on our ***S***_***g***_ statistic**. Significance was defined by *p* < 0.05 after 1000 permutations. Each of the four independent analyses is based on different soft thresholds ranging from 3 (PERM.SP3) to 6 (PERM.SP6).

We had previously found interesting differences in chromosome copy number in several genomic intervals when comparing groups with different TP53 mutation types in high grade serous ovarian cancer samples, and in that work we also found that the TP53 mutation types showed association with overall/progression free survival (Wojnarowicz et al., 2012). Therefore, we first calculated our *S*_*g*_ statistics in the same histological subtype of TP53 mutation-positive ovarian cancer samples from the TCGA data. We analyzed 14,939 genes after filtering to remove genes with low variability, selected from 19,919 genes reported in the RNA-sequencing data. Permutation analyses (1000 permutations) were undertaken to assess statistical significance of the differential coexpression for each gene and for a range of soft thresholds, and then we examined overlap between the genes with unadjusted empirical *p* < 0.05 across the soft threshold range. Figure 3 shows that although over 300 genes were identified with *p* < 0.05 in each of the analyses with soft thresholds between 3 and 6 (range 327–378 genes), there is a core set of 176 genes that consistently display significantly distinct relationships (or network connectivity) in the tumor groups parsed according to TP53 missense or null mutation type.

Using these 176 genes showing differential coexpression at all soft thresholds, we performed gene set enrichment analysis to begin to identify molecular pathways/processes that could distinguish the two TP53 mutation-type tumor groups. We identified a few KEGG pathways, such as pyrimidine and purine metabolism and DNA replication (Supplemental Table S2), and GO terms associated with metabolic processes, telomere maintenance and DNA replication (Supplemental Table S3) that appeared to be over-represented (adjusted *p* < 0.05).

We then repeated the same analytic pipeline for triple negative breast cancer, skin cutaneous melanoma (amongst which most tumors were metastatic), and lung adenocarcinoma in order to verify our initial findings with the ovarian cancer set. Using the same method, we identified the genes that showed statistically significant evidence for differential coexpression at all soft thresholds between our low and high limits (Table 1), for each cancer. The number of genes identified as significant showed limited variability across the soft thresholds within a cancer type, but varied quite substantially across different cancers, with over 1200 significant genes for SKCM and closer to 300 for OV and LUAD.

### Finding communalities in differential coexpression among cancer types

We then examined the overlap between the genes identified in our analyses of each cancer type to search for pathways that might indicate some communalities in the differential coexpression networks based on TP53 mutation type. Only one gene, KIR3DL2, displayed prominent differential coexpression across all cancers and soft thresholds. For three cancers (OV, BRCA, LUAD), differential coexpression was nominally significant (*p* < 0.05) for all soft thresholds. For our fourth cancer, SKCM, KIR3DL2 showed significant evidence (nominal *p* < 0.05) for differential coexpression with 3 of the 4 soft thresholds analyzed, and was non-significant at the threshold of 0.05 (nominal *p* = 0.069) for the fourth soft threshold. It should be noted that power was lowest for the analysis of SKCM since there were only 59 cancer samples harboring TP53 mutations that were available for our analyses, in contrast to the larger data sets available for each of the other cancer types which ranged from 80 to 260 samples (Table 1).

**Figure 4:**
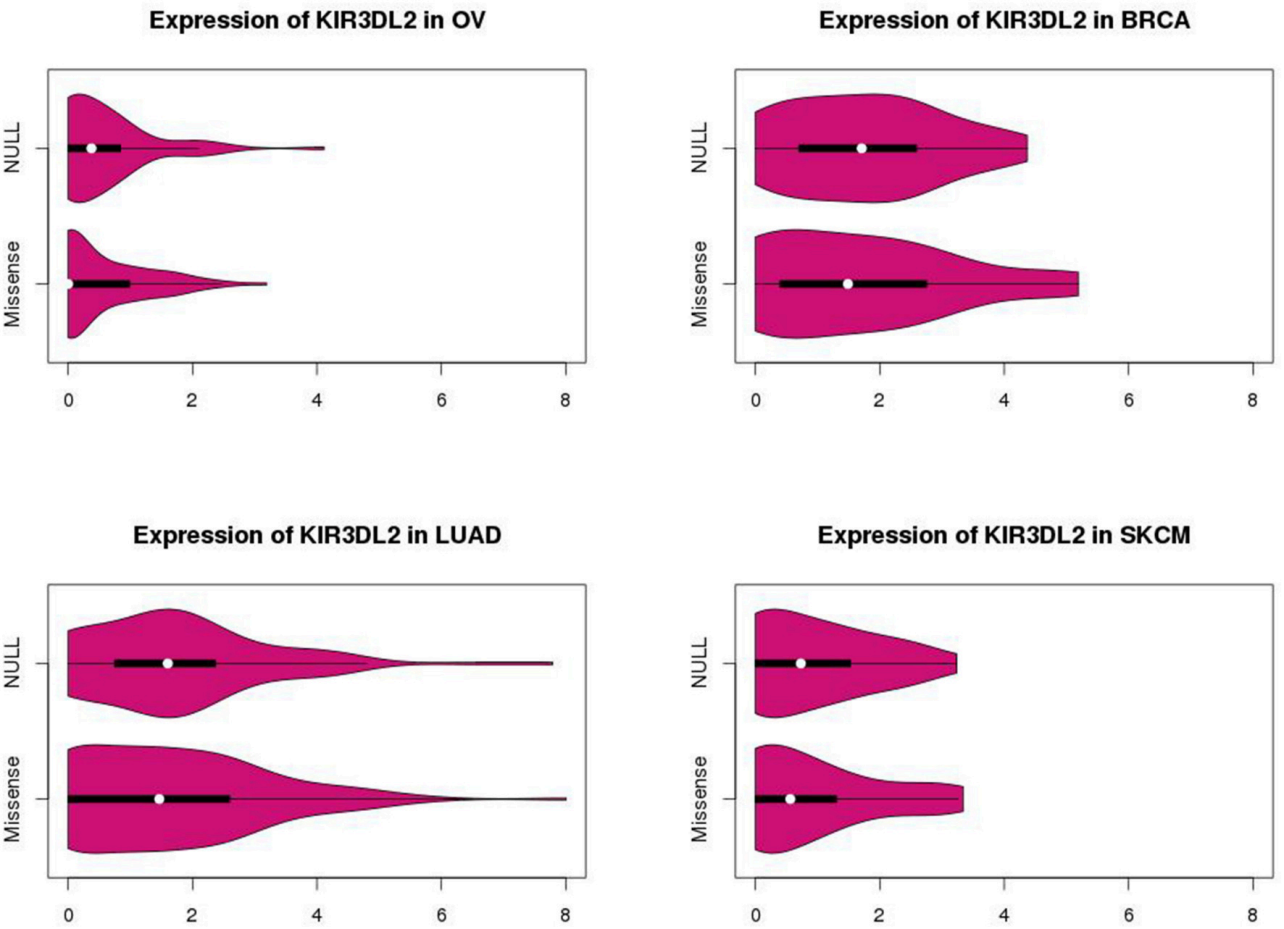
**Violin plots showing expression distributions for KIR3DL2 by p53 missense or null mutation type in ovarian (OV), breast (BRCA), and lung (LUAD) cancers and skin melanomas (SKCM)**. *P*-values of 0.62 for OV, 0.45 for BRCA, 0.46 for LUAD, and 0.73 for SKCM were obtained from differential expression analysis comparing KIR3DL2 expression levels between p53 null and missense tumors.

**Figure 5:**
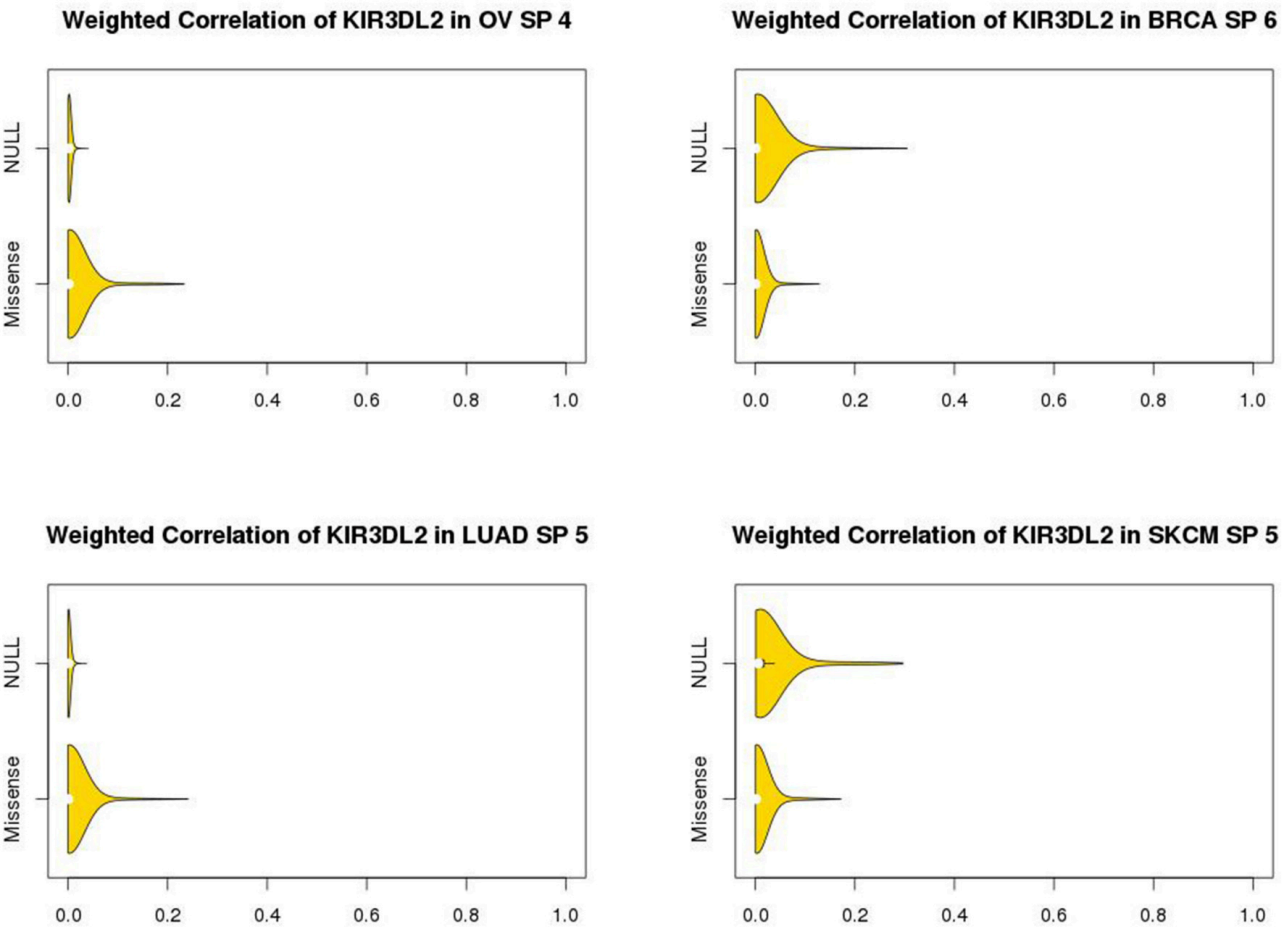
**Violin plots showing coexpression distributions for KIR3DL2 by p53 missense or null mutation type in ovarian (OV), breast (BRCA), and lung (LUAD) cancers and skin melanomas (SKCM)**. Distributions of the absolute values of overlap measures between KIR3DL2 and all other genes, using the optimized soft-threshold value for each cancer type. The soft thresholds used were 4 for OV, 6 for BRCA, 5 for LUAD, and 5 for SKCM. With these soft thresholds, there was evidence of statistically significant differences in coexpression of KIR3DL2 based on permutation analysis: *P*-values were 0.02 for OV, 0.03 for BRCA, 0.002 for LUAD and 0.05 for SKCM.

The marginal expression levels of KIR3DL2 vary from one cancer type to another (Figure 4). For example, there is very little expression in OV, but a long-tailed distribution in LUAD. However, there is no evidence for differential expression between the p53 null and missense tumors (Figure 4): univariate tests for differential expression of KIR3DL2 using the Bioconductor package “limma” (Smyth, 2004) were non-significant. In contrast, the absolute values of the overlap measures between KIR3DL2 and other genes show distinct distributions depending on the TP53 mutation type (Figure 5). Using our optimal soft threshold for each cancer, the empirical *p*-values testing for differences in coexpression at KIR3DL2 were 0.02 for OV, 0.03 for BRCA, 0.002 for LUAD, and 0.05 for SKCM.

For OV and LUAD, tumors with missense mutations in TP53 tended to show stronger coexpression between KIR3DL2 and other genes, whereas the tumors with null mutations in TP53 showed stronger coexpression in BRCA and SKCM.

### Finding differentially-coexpressed genes

To find the key partner genes of KIR3DL2, i.e., the genes with the most differential coexpression between TP53 mutation types, we extracted the genes that had overlap measures greater than “expected” for the optimized soft-thresholds. In this context, we defined expected thresholds, separately for each cancer, as the maximum overlap measure in the group with lower coexpression range in Figure 5. For OV and LUAD this was the maximum of the range of the overlap measure found in the tumors with null mutations (0.04), whereas we defined the expected range from tumors with missense mutations for BRCA (maximum 0.13) and SKCM (maximum 0.17). After finding these thresholds, we then selected genes with overlap measures higher than these cutoffs in the other mutation-type group.

**Figure 6:**
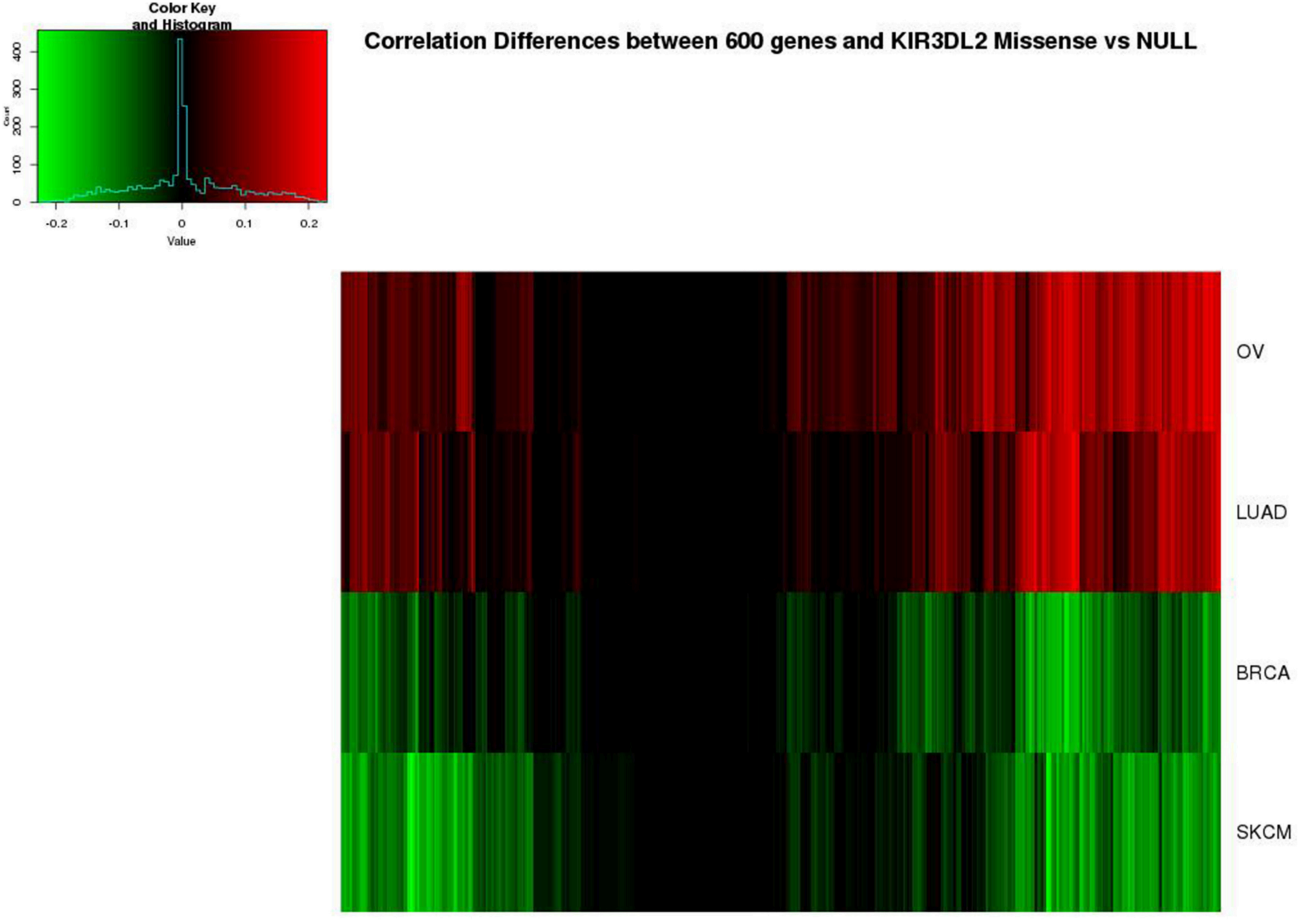
**Heatmap of differences (missense − null) in overlap measures between KIR3DL2 and 600 genes with strong connections to this gene in OV**. The same 600 genes are shown for all four cancers, in the same order.

Using the above selection criteria, we selected 600 genes with strong overlap measures between KIR3DL2 and other genes in OV. In Figure 6, the differences (*T*_*gjm*_ − *T*_*gjn*_) are shown for *g* = *KIR*3*DL*2 and these ovarian-cancer selected genes, *j* = 1, … 600, but these differences are shown for all four cancers. Evidently, the missense-null differences in overlap measure vary substantially across cancers, even though many of the same partner genes are highlighted in more than one cancer.

After repeating a similar selection of genes for all four cancers, we examined the overlap among the selected gene lists across the four cancers, and identified 164 genes that were differentially correlated to KIR3DL2 in all four cancer types. KEGG pathway analyses of these 164 genes implicated 37 significant pathways (Table 2), and it is interesting to note that many immune-related pathways appear to show evidence of differential overlap measures with our core gene KIR3DL2. In contrast, when we examined these 164 genes for evidence of changes in the mean levels of expression between the missense and null mutation-carrying tumors, no particular patterns are detectable. Supplemental Figure S2 shows a heatmap simply of expression levels for OV at these 164 genes indicating no clustering of the two mutation types; the other cancer types show similar patterns.

**Table 2:** **Significantly enriched KEGG pathways resulting from the analysis of 164 genes differentially correlated to KIR3DL2 between p53 missense and null mutation groups for all cancer types**.

**ID**	**Description**	**GeneRatio^a^**	**BgRatio^b^**	***P*-value^c^**	**p.adjust^d^**	***Q*-value^e^**
hsa04514	Cell adhesion molecules (CAMs)	20/89	142/6971	4.71E-16	6.13E-14	4.57E-14
hsa05330	Allograft rejection	12/89	37/6971	1.25E-14	8.12E-13	6.05E-13
hsa04940	Type I diabetes mellitus	12/89	43/6971	9.72E-14	4.21E-12	3.14E-12
hsa05340	Primary immunodeficiency	11/89	36/6971	3.61E-13	1.17E-11	8.74E-12
hsa05332	Graft-vs.-host disease	11/89	41/6971	1.80E-12	4.69E-11	3.49E-11
hsa04660	T cell receptor signaling pathway	15/89	104/6971	2.21E-12	4.79E-11	3.57E-11
hsa04060	Cytokine-cytokine receptor interaction	21/89	265/6971	8.81E-12	1.64E-10	1.22E-10
hsa05320	Autoimmune thyroid disease	11/89	52/6971	3.08E-11	5.00E-10	3.72E-10
hsa05416	Viral myocarditis	11/89	58/6971	1.09E-10	1.57E-09	1.17E-09
hsa05321	Inflammatory bowel disease (IBD)	11/89	65/6971	3.98E-10	5.18E-09	3.86E-09
hsa04650	Natural killer cell mediated cytotoxicity	14/89	134/6971	1.06E-09	1.26E-08	9.36E-09
hsa04672	Intestinal immune network for IgA production	9/89	47/6971	5.47E-09	5.93E-08	4.42E-08
hsa05150	Staphylococcus aureus infection	9/89	55/6971	2.35E-08	2.35E-07	1.75E-07
hsa04612	Antigen processing and presentation	10/89	77/6971	3.75E-08	3.48E-07	2.59E-07
hsa04640	Hematopoietic cell lineage	10/89	87/6971	1.23E-07	1.07E-06	7.96E-07
hsa05323	Rheumatoid arthritis	10/89	89/6971	1.53E-07	1.25E-06	9.28E-07
hsa05145	Toxoplasmosis	11/89	118/6971	2.46E-07	1.88E-06	1.40E-06
hsa04062	Chemokine signaling pathway	13/89	187/6971	5.80E-07	4.19E-06	3.12E-06
hsa05142	Chagas disease (American trypanosomiasis)	10/89	104/6971	6.74E-07	4.61E-06	3.43E-06
hsa05322	Systemic lupus erythematosus	11/89	134/6971	8.94E-07	5.81E-06	4.33E-06
hsa05310	Asthma	6/89	30/6971	1.70E-06	1.05E-05	7.82E-06
hsa05166	HTLV-I infection	14/89	258/6971	4.04E-06	2.39E-05	1.78E-05
hsa05162	Measles	10/89	134/6971	6.89E-06	3.89E-05	2.90E-05
hsa05140	Leishmaniasis	7/89	72/6971	3.28E-05	1.77E-04	1.32E-04
hsa05164	Influenza A	10/89	175/6971	7.04E-05	3.66E-04	2.73E-04
hsa05152	Tuberculosis	10/89	177/6971	7.75E-05	3.87E-04	2.89E-04
hsa05168	Herpes simplex infection	10/89	184/6971	1.07E-04	5.16E-04	3.84E-04
hsa05144	Malaria	5/89	49/6971	3.71E-04	1.72E-03	1.28E-03
hsa04630	Jak-STAT signaling pathway	8/89	158/6971	8.69E-04	3.90E-03	2.90E-03
hsa05169	Epstein-Barr virus infection	10/89	200/6971	9.61E-04	4.16E-03	3.10E-03
hsa04380	Osteoclast differentiation	7/89	131/6971	1.35E-03	5.67E-03	4.22E-03
hsa04145	Phagosome	7/89	153/6971	3.27E-03	1.33E-02	9.90E-03
hsa04670	Leukocyte transendothelial migration	6/89	118/6971	3.84E-03	1.51E-02	1.13E-02
hsa04666	Fc gamma R-mediated phagocytosis	5/89	92/6971	6.23E-03	2.38E-02	1.78E-02
hsa05020	Prion diseases	5/89	35/6971	9.80E-03	3.64E-02	2.71E-02

a *GeneRatio: # significant genes in the pathway/# significant genes*.

b *BgRatio: # genes in pathway/# genes in all pathways*.

c *P-value: Test of enrichment for pathways based on the hypergeometric distribution*.

d *p.adjust: Benjamini and Hochberg adjusted p-value (Benjamini and Hochberg, 1995)*.

e *Q-value: False discovery rate (Storey, 2002)*.

## Discussion

High throughput differential gene expression analysis has been able to identify and re-affirm the importance of specific molecular pathways in cancers over the last 20 years. A 2003 review (Liang and Pardee, 2003) predicted substantial potential for the technology, and today this is illustrated by the vast array of publications resulting from TCGA endeavors (https://tcga-data.nci.nih.gov/docs/publications/) describing expression profiles of numerous cancer types in detail. A consistent observation across independent studies is the significant heterogeneity observed by the analysis of gene expression profiles even among cancers classified by histological subtype. Thus a major focus of research has been (and is) using gene expression profiling data for subtype classification of cancers with the eventual goal of enabling the development or re-purposing of therapies suitable for each subtype. For example, TCGA reported significant heterogeneity among the high grade serous ovarian carcinomas profiled for gene expression, where they used (non-negative matrix factorization-based) clustering to delineate at least four transcriptional subtypes (Cancer Genome Atlas Research, 2011), which was subsequently validated in a subsequent study that highlighted the therapeutic relevance of the molecular subtypes (Konecny et al., 2014). The significance of these findings are currently unknown, however could explain in part the heterogeneity in response to first-line therapeutics observed for women with high grade serous ovarian cancer (Ovarian Epithelial, Fallopian Tube, and Primary Peritoneal Cancer Treatment–for health professionals (PDQ®), http://www.cancer.gov/types/ovarian/hp/ovarian-epithelial-treatment-pdq). The translational potential of any of the gene expression signatures to predict survival and subtype in clinical settings is currently uncertain, though promising results have been demonstrated when applied to fresh and paraffin embedded ovarian tumors (Sfakianos et al., 2013). However the potential of molecular subtyping to guide therapy decision has yet to be realized not only due to the limited arsenal for therapeutics currently available for this disease but because questions remain about the consistency and interpretability of gene-expression-based signatures as a clinical tool.

However, different analytic perspectives can often lead to new insights especially when combined with additional molecular genetic information such as the nature of TP53 mutation. Here, we have proposed a test statistic that highlights differential coexpression between two groups defined by the presence of TP53 missense and null mutations, and hence finding genes that show very different patterns of the overlap measures (built on the weighted correlations) and connectivity. This approach, combined with the sets of genes that we identified, provides suggestive evidence that genes are being regulated and controlled differently in the two mutation groups in several different cancers.

As mentioned in the Introduction, several authors have addressed the question of how to compare coexpression patterns between groups. One proposed method (Miller et al., 2010), used WGCNA to cluster the TOM matrices separately for the two groups, and then examined the overlap in cluster membership. Another WGCNA-derived method (Tesson et al., 2010), was designed to find modules of genes showing differential coexpression. In contrast to both these papers, our perspective here is distinct, since we have created a measure of differential coexpression for each gene (*S*_*g*_), allowing us to rank individual genes with respect to their differences in connectivity, or said differently, to look for whether some genes have varying roles in coordinating coexpression. We find it interesting to note that single genes display quite different evidence of connectivity between our two mutation groups, and this feature is not highlighted in the same way by a clustering analysis. When comparing our approach to (Dettling et al., 2005) and (Hu R. et al., 2009), we have worked with the TOM matrices rather than with the pairwise correlations. The former identifies the gene pairs that show evidence of large differences between groups, and they use permutation of samples to obtain a null distribution for their gene pair difference measure. In the latter, the *N* statistic of (Hu P. et al., 2009) compares the distributions of correlations between the two groups to the distributions of correlations within each of these groups. This requires either resampling or subgrouping in order to create sets of correlations that can be jointly examined. The idea of examining the joint distribution of correlations, which is encapsulated in this *N*-statistic, is important since it provides a more global view of the relationships. In fact, the overlap measure (Ravasz et al., 2002) used in WGCNA can be considered to have the same goal, since the measure of similarity for each pair of genes is calculated as a function of the connections between all other genes and each member of the pair.

Our direct use of the TOM matrix for calculating differences in overlap measures required an investigation into the impact of the soft threshold parameter, and we found that the distribution of the test statistics *S*_*g*_ was quite sensitive to this tuning parameter. Simulations showed better agreement among the significant results when the scale-free network assessment showed adequate goodness of fit (see Supplemental Methods). For assessing statistical significance, we performed 1000 permutations and re-analyzed the data with the same soft thresholds, to find sets of genes, at each value of β, that showed empirical *p* < 0.05. Naively, given that we analyzed over 14,000 genes, one might expect ~700 genes to show significance by chance at significance level α = 0.05 (for one soft threshold), and in fact we found fewer than this. Although we have analyzed each cancer data set with a range of soft thresholds, if the optimal choice of β differs between the groups, then the permuted data will not be optimally transformed, and this is likely to influence the test statistic distribution and hence, the power.

One popular way to improve power in gene expression studies is achieved through restricting analysis to a smaller set of genes, and hence alleviating the multiple testing adjustment. Gene filtering is often based on variability in gene expression—as we have done—although many other statistically-motivated strategies have been proposed (Lazar et al., 2012). Alternatively, analysis can be restricted to genes that are thought be plausible candidates, based on external information. In our study, although quite a bit is known about differences in gene expression between wild type and mutant p53 (e.g., O'Farrell et al., 2004), no previous exploration has been undertaken of differences in expression in cancer cells expressing mutant p53 proteins vs. no protein. Furthermore, the coexpression behaviors are even less well-understood. In order to maximize the set of possibly-coexpressed genes, we decided not to further restrict our gene set prior to analysis. This decision may have reduced our power.

Nevertheless, even if power for our approach is low, we have taken care to implement a stringent approach for protecting our results against false positives by selecting only genes showing significance across all the soft thresholds used to analyze each cancer type, and across all four cancers. We undertook a small simulation study to obtain a rough estimate of our overall type 1 error rate, built around the results in Table 1. Specifically, we generated multivariate normal deviates for 14,800 genes, four cancers and 4 or 5 soft thresholds (following Table 1), assuming dependence between the normal deviates across soft thresholds with correlation = 0.8, but independence across genes and cancers. We then transformed the *z*-values to *p*-values, and applied significance thresholds in line with Table 1 assuming a uniform distribution (i.e., *p* ≤ 350/14,800 for OV, *p* ≤ 930/14800 for BRCA, *p* ≤ 1200/14,800 for SKCM, *p* ≤ 370/14,800 for LUAD). In none of 1000 simulations was the same gene selected in all four cancers and all soft thresholds. While recognizing the limitations of this simple simulation, it makes a strong case that our identification of KIR3DL2 is very unlikely to occur by chance.

KIR3DL2 shows evidence of very different coexpression patterns between tumors carrying TP53 null and missense mutations. This gene and its associated network would not have been identified by a traditional differential expression analysis, as was demonstrated in Figure 4.

KIR3DL2, a member of a large and complex gene family, encodes killer cell immunoglobulin-like receptors (KIR) that are expressed by natural killer cells and subsets of T lymphocytes, which modulate their effector functions through binding to their cognate MHC class I ligands (Benson and Caligiuri, 2014). KIR3DL2's role in the regulation of the immune response is intriguing in light of the tissue specific expression, and perhaps the identification of this gene using our method is an indication of the extent of the involvement of immune cells in tumor samples. Thus, although it is of interest to examine the potential role of KIR3DL2 in differentiating the mutation types, given our analysis of coexpression it is more interesting to examine the set of genes which show the most evidence of different correlations with KIR3DL2 in the two mutation type groups. Pathway analyses of the 164 genes showing the most alterations in correlations led to evidence of enrichment of a number of immune-related pathways (Table 2). These findings are intriguing, as it is increasingly becoming apparent that the tumor microenvironment with respect to cells and molecules of the immune system are important in the biology of cancer contributing to the tumor initiation, tumor progression and response to therapy (see Nature Reviews Rheumatology http://www.nature.com/reviews/focus/tumourimmunology/index.html and references therein for this special focus). Moreover, a number of studies have shown that a variety of solid tumors exhibit evidence of T-cell infiltration, chemokines and an interferon profile indicative of innate immune activation by the host, which likely result in resistance of immune attack through the dominant inhibitory effects of immune system–suppressive pathways (reviewed in Gajewski et al., 2013). This phenotype differs from cancer cells that lack this T-cell inflamed phenotype, which appear to resist immune attack. These phenotypes have been associated with response to therapy and overall outcome (reviewed in Gajewski et al., 2013). Although the role of p53 in regulating immune-regulating gene expression networks has been the subject of intense study, less is known about the role of mutant p53 isoforms in this context. However, there is evidence in cancer cell model systems that mutant p53 can modulate the expression of immunoregulatory genes (reviewed in Menendez et al., 2013). Further research associating the major immune-phenotypes observed in solid tumors with TP53 mutation type rather than status alone might clarify the role of mutant p53 isoforms in tumor cell microenvironment. Our implication of differential coexpression between TP53 mutation types should perhaps be considered when studying the role of the immune system.

We chose four different cancer types for analysis where TP53 is fairly commonly mutated, but otherwise having very different characteristics. Lung adenocarcinoma and (metastatic) melanoma are largely attributable to environmental exposures, and occur in both men and women. In contrast, ovarian cancers are restricted to women, triple negative breast cancer occurs primarily in women, and the role of environment is much smaller. Our unique analytical approach as applied to these diverse cancers grouped according to TP53 mutation type has provided evidence for a new subgroup classification that warrants further investigation. The overall prognosis is poor for all of histological types of cancer investigated in this study, and hence an improved understanding of the biology of these cancers will be of great benefit as the findings are translated into clinical settings.

## Conclusions

Considering patterns of coexpression in tumors carrying either missense or null mutations in TP53 is a fruitful strategy that leads to a set of genes showing evidence for differential coexpression and enriched for many immune system pathways.

## Author contributions

KOK performed all analyses of the tumors, and assisted with writing the manuscript. KO supervised the simulations relating to soft thresholds and participated in the conception of the study. ML performed the simulations relating to soft thresholds. SB contributed to the concept. PT, who is co-senior author, motivated the study participated in conception of the design, and wrote part of the manuscript. CG conceived the study, proposed the test statistic, supervised the analysis, and wrote most of the manuscript.

## Funding

Funding was provided by les Fonds de Recherche du Québec - Santé (#31110), the Weekend to End Women's Cancers and the Ludmer Centre for Neuroinformatics and Mental Health.

### Conflict of interest statement

The authors declare that the research was conducted in the absence of any commercial or financial relationships that could be construed as a potential conflict of interest. The reviewer RK and handling Editor declared their shared affiliation, and the handling Editor states that the process nevertheless met the standards of a fair and objective review.
