# Supplementary material for: Gene Coexpression Analyses Differentiate Networks Associated with Diverse Cancers Harboring TP53 Missense or Null Mutations
Source: Front Genet. 2016 Aug 3;7:137. doi: 10.3389/fgene.2016.00137 (PMC4971393; doi:10.3389/fgene.2016.00137)
Supplement: Supplementary file 1 [file DataSheet1.docx]

**SUPPLEMENT**

**Gene co-expression analyses differentiate networks associated with diverse cancers harbouring TP53 missense or null mutations**

**Kathleen Oros Klein, Karim Oualkacha, Marie-Hélène Lafond, Sahir Bhatnagar, Patricia N. Tonin, Celia M.T. Greenwood**

**Table of Contents**

1. Supplemental Methods
2. Supplemental Figure S1
3. Supplemental Figure S2
4. Supplemental Table Legends
5. Supplemental Table S2
6. Supplemental Table S3
7. **Supplemental Methods**

Parameters in the simulation to assess the impact of the soft threshold on $S_{g}$ were as follows. There were 50 tumors divided into two groups of 25 tumors each (e.g. two different mutation type groups such as p53 missense or null mutations). There were 3000 genes divided into 6 correlated clusters of the following sizes: 450, 240, 120, 1410, 600, and 180 genes, and correlation patterns in two of these clusters were differentially associated with the group status. Specifically, this was implemented so that the eigenvector capturing expression patterns showed strong negative correlation (-0.6) between the cluster of 240 genes and group status, and a strong positive correlation (+0.6) between the cluster of 120 genes and the group status. The remaining clusters of genes were not associated with the grouping variable. Most clusters were uncorrelated with each other, but there was correlation between the expression levels of genes in the clusters of size 450 and 600. Supplement Figure S1 shows that skewness increases with the soft threshold. Higher values better identify the genes in the simulated differentially-correlated clusters, and this result held true in simulations not only for our $S_{g}$ statistic, but also for logistic models, the LASSO^1^ , GGLASSO^2^ and SparseLDA^3^.

^1^ Tibshirani, R (1996). Regression shrinkage and selection via the lasso. J Royal Statist Soc Series B 58: 267 – 288.

^2^ Yang, Y, Zou H (2015). A fast unified algorithm for solving group-lasso penalize learning problems. Statistics and Computing 25(6) 1129-1141.

^3^ Clemmensen L, Hastie T, Witten D, and Ersbøll B (2011). Sparse discriminant analysis. Technometrics, 53(4): 406-413.

1. **Supplement Figure S1. Histogram of S_g values for soft thresholds of 1, 3, 4 and 7.**


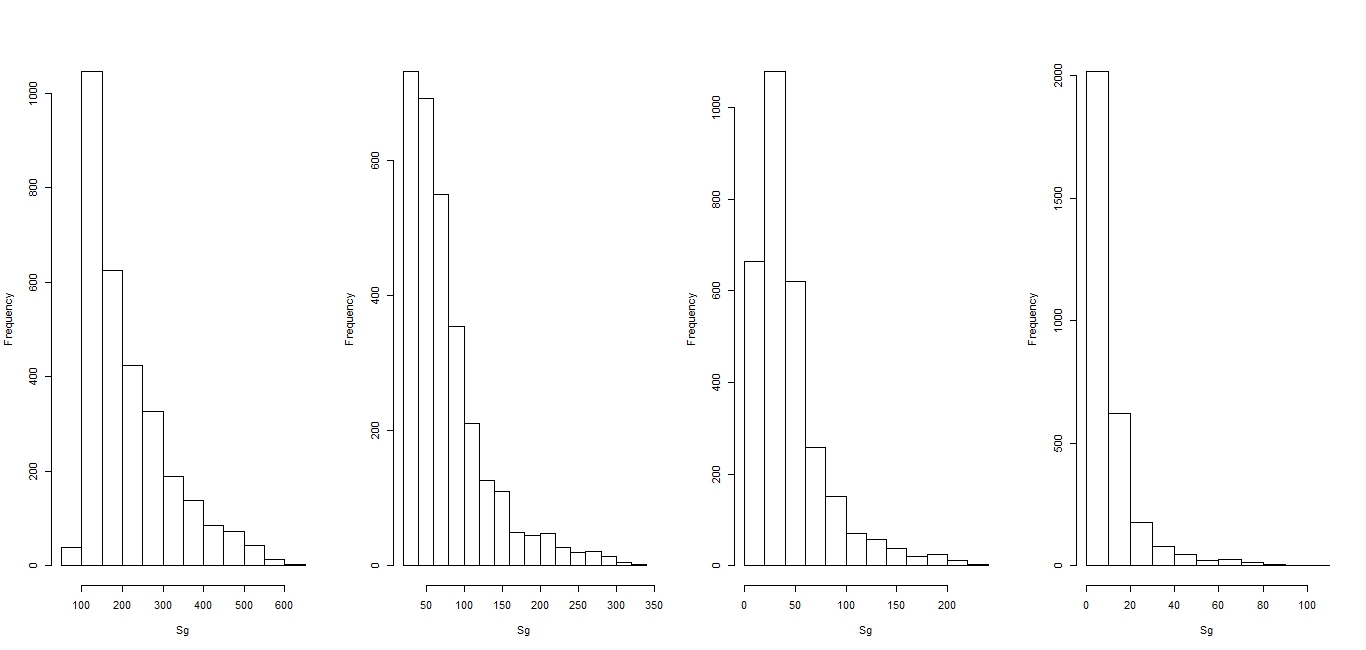


1. **Supplemental Figure S2.** Heatmap of expression in OV for 164 genes that show differential co-expression with KIR3DL2. The absolute levels of expression are shown. The colored axis on the top indicates the mutation type in the tumors, with missense in blue and null in

**
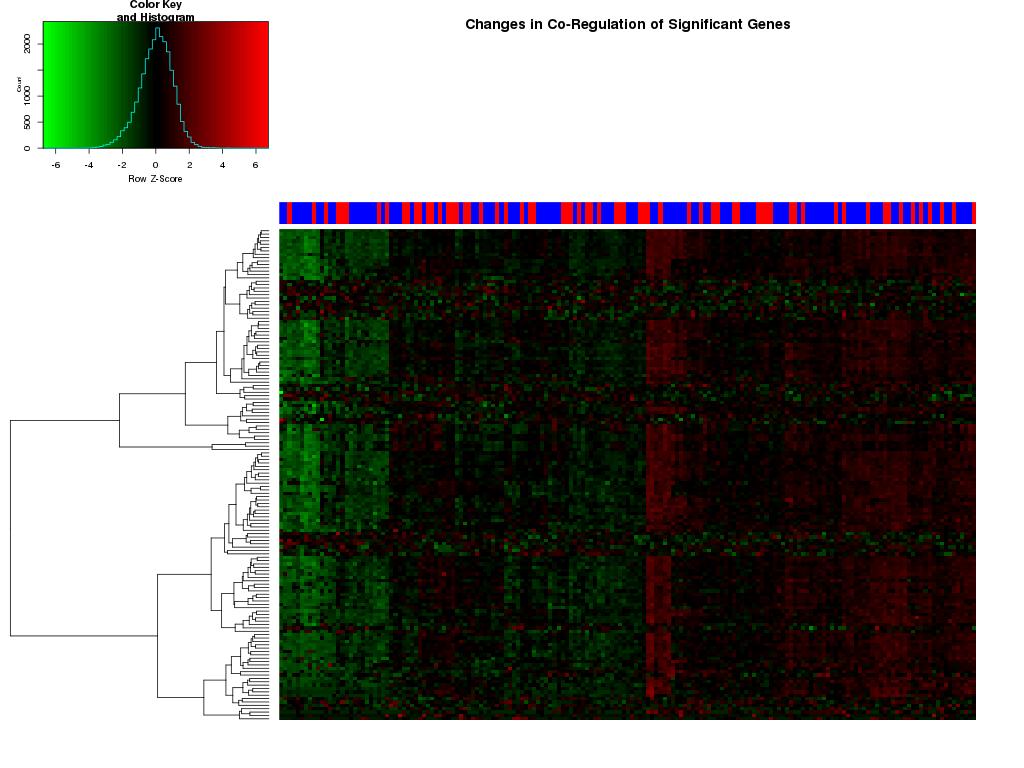
** red.

1. **Supplemental Table Legends**

**Supplement Table S1.** List of all RNA-seq files obtained from TCGA. See separate attachment

PT.TCGA.Downloaded_Files_IlluminaHiSeq_RNASeqV2.txt

**Supplement Table S2.** Significant KEGG pathways for differentially co-expressed genes in ovarian cancer tumors with p53 null or missense mutations groups

**Supplement Table S3.** Significant GO biological processes for differentially correlated genes in ovarian cancer tumors with p53 null or missense mutations groups

1. **Supplement Table S2. Significant KEGG pathways for differentially co-expressed genes in ovarian cancer tumors with null or missense mutations in TP53.**

| ID | Description | GeneRatio | BgRatio | pvalue | p.adjust | qvalue |
| --- | --- | --- | --- | --- | --- | --- |
| hsa00240 | Pyrimidine metabolism | 07/58 | 105/6971 | 2.30E-05 | 0.003015 | 0.002811 |
| hsa00230 | Purine metabolism | 08/58 | 176/6971 | 9.19E-05 | 0.006016 | 0.005608 |
| hsa03030 | DNA replication | 04/58 | 36/6971 | 2.08E-04 | 0.009101 | 0.008483 |
| hsa05016 | Huntington's disease | 07/58 | 193/6971 | 1.02E-03 | 0.033315 | 0.031053 |

GeneRatio: # significant genes in the pathway / # significant genes

BgRatio: # genes in pathway/# genes in all pathways

Pvalue: Test of enrichment for pathways based on the hypergeometric distribution

p.adjust: Benjamini and Hochberg adjusted p-value Benjamini, Y, Hochberg, Y (1995). Controlling the false discovery rate: a practical and powerful approach to multiple testing. JRSS B 57: 289-300.

qvalue: False discovery rate, Storey 2002. A direct approach to false discovery rates. JRSS B 64: 479-198.

1. **Supplement Table S3. Significant GO biological processes for differentially correlated genes in ovarian cancer tumors with null or missense mutations in TP53.**

| ID | Description | GeneRatio | BgRatio | pvalue | p.adjust | qvalue |
| --- | --- | --- | --- | --- | --- | --- |
| GO:0008150 | biological_process | 143/143 | 11335/12852 | 1.42E-08 | 2.87E-05 | 2.72E-05 |
| GO:0046483 | heterocycle metabolic process | 69/143 | 3439/12852 | 2.79E-08 | 2.87E-05 | 2.72E-05 |
| GO:0006725 | cellular aromatic compound metabolic process | 69/143 | 3462/12852 | 3.72E-08 | 2.87E-05 | 2.72E-05 |
| GO:0006139 | nucleobase-containing compound metabolic process | 66/143 | 3298/12852 | 8.36E-08 | 4.63E-05 | 4.39E-05 |
| GO:1901360 | organic cyclic compound metabolic process | 70/143 | 3631/12852 | 1.13E-07 | 4.63E-05 | 4.39E-05 |
| GO:0034641 | cellular nitrogen compound metabolic process | 72/143 | 3794/12852 | 1.20E-07 | 4.63E-05 | 4.39E-05 |
| GO:0010833 | telomere maintenance via telomere lengthening | 6/143 | 23/12852 | 1.48E-07 | 4.87E-05 | 4.61E-05 |
| GO:0006807 | nitrogen compound metabolic process | 73/143 | 4030/12852 | 6.73E-07 | 1.94E-04 | 1.84E-04 |
| GO:0000723 | telomere maintenance | 7/143 | 52/12852 | 1.60E-06 | 3.91E-04 | 3.71E-04 |
| GO:0044271 | cellular nitrogen compound biosynthetic process | 57/143 | 2886/12852 | 1.98E-06 | 3.91E-04 | 3.71E-04 |
| GO:0032200 | telomere organization | 7/143 | 54/12852 | 2.08E-06 | 3.91E-04 | 3.71E-04 |
| GO:0022616 | DNA strand elongation | 5/143 | 20/12852 | 2.15E-06 | 3.91E-04 | 3.71E-04 |
| GO:0090304 | nucleic acid metabolic process | 57/143 | 2895/12852 | 2.20E-06 | 3.91E-04 | 3.71E-04 |
| GO:0034645 | cellular macromolecule biosynthetic process | 57/143 | 2908/12852 | 2.55E-06 | 4.22E-04 | 4.00E-04 |
| GO:0009059 | macromolecule biosynthetic process | 58/143 | 3005/12852 | 3.27E-06 | 5.04E-04 | 4.78E-04 |
| GO:0044237 | cellular metabolic process | 97/143 | 6354/12852 | 5.93E-06 | 8.36E-04 | 7.92E-04 |
| GO:0071704 | organic substance metabolic process | 100/143 | 6641/12852 | 6.15E-06 | 8.36E-04 | 7.92E-04 |
| GO:0032201 | telomere maintenance via semi-conservative replication | 4/143 | 12/12852 | 6.79E-06 | 8.71E-04 | 8.26E-04 |
| GO:0044238 | primary metabolic process | 97/143 | 6386/12852 | 7.78E-06 | 9.45E-04 | 8.96E-04 |
| GO:0018130 | heterocycle biosynthetic process | 52/143 | 2655/12852 | 9.66E-06 | 1.12E-03 | 1.06E-03 |
| GO:0019438 | aromatic compound biosynthetic process | 52/143 | 2664/12852 | 1.07E-05 | 1.18E-03 | 1.11E-03 |
| GO:0016070 | RNA metabolic process | 51/143 | 2599/12852 | 1.18E-05 | 1.23E-03 | 1.17E-03 |
| GO:0034654 | nucleobase-containing compound biosynthetic process | 51/143 | 2610/12852 | 1.33E-05 | 1.33E-03 | 1.27E-03 |
| GO:0032774 | RNA biosynthetic process | 47/143 | 2341/12852 | 1.71E-05 | 1.62E-03 | 1.54E-03 |
| GO:0044260 | cellular macromolecule metabolic process | 80/143 | 4962/12852 | 1.81E-05 | 1.62E-03 | 1.54E-03 |
| GO:0000722 | telomere maintenance via recombination | 4/143 | 15/12852 | 1.82E-05 | 1.62E-03 | 1.54E-03 |
| GO:1901362 | organic cyclic compound biosynthetic process | 52/143 | 2762/12852 | 3.05E-05 | 2.61E-03 | 2.47E-03 |
| GO:0043170 | macromolecule metabolic process | 85/143 | 5493/12852 | 3.93E-05 | 3.17E-03 | 3.01E-03 |
| GO:0006271 | DNA strand elongation involved in DNA replication | 4/143 | 18/12852 | 3.98E-05 | 3.17E-03 | 3.01E-03 |
| GO:0009987 | cellular process | 130/143 | 10054/12852 | 4.57E-05 | 3.52E-03 | 3.33E-03 |
| GO:0033260 | nuclear DNA replication | 4/143 | 21/12852 | 7.59E-05 | 5.66E-03 | 5.36E-03 |
| GO:0008152 | metabolic process | 106/143 | 7564/12852 | 9.20E-05 | 6.62E-03 | 6.27E-03 |
| GO:0051276 | chromosome organization | 17/143 | 536/12852 | 9.45E-05 | 6.62E-03 | 6.27E-03 |
| GO:0006351 | transcription, DNA-templated | 44/143 | 2298/12852 | 1.15E-04 | 7.80E-03 | 7.39E-03 |
| GO:0097659 | nucleic acid-templated transcription | 44/143 | 2304/12852 | 1.22E-04 | 8.06E-03 | 7.64E-03 |
| GO:0006260 | DNA replication | 9/143 | 174/12852 | 1.40E-04 | 8.99E-03 | 8.52E-03 |
| GO:0034728 | nucleosome organization | 7/143 | 103/12852 | 1.48E-04 | 9.26E-03 | 8.77E-03 |
| GO:0044786 | cell cycle DNA replication | 4/143 | 25/12852 | 1.55E-04 | 9.33E-03 | 8.84E-03 |
| GO:0031497 | chromatin assembly | 7/143 | 104/12852 | 1.58E-04 | 9.33E-03 | 8.84E-03 |
| GO:1901576 | organic substance biosynthetic process | 62/143 | 3727/12852 | 1.66E-04 | 9.57E-03 | 9.07E-03 |
| GO:0044249 | cellular biosynthetic process | 61/143 | 3649/12852 | 1.70E-04 | 9.57E-03 | 9.07E-03 |
| GO:0006312 | mitotic recombination | 4/143 | 26/12852 | 1.82E-04 | 9.99E-03 | 9.47E-03 |

GeneRatio: # significant genes in the pathway / # significant genes

BgRatio: # genes in pathway/# genes in all pathways

Pvalue: Test of enrichment for pathways based on the hypergeometric distribution

p.adjust: Benjamini and Hochberg adjusted p-value Benjamini, Y, Hochberg, Y (1995). Controlling the false discovery rate: a practical and powerful approach to multiple testing. JRSS B 57: 289-300.

qvalue: False discovery rate, Storey 2002. A direct approach to false discovery rates. JRSS B 64: 479-198.
