# Supplementary material for: Gene Coexpression Analyses Differentiate Networks Associated with Diverse Cancers Harboring TP53 Missense or Null Mutations
Source: Front Genet. 2016 Aug 3;7:137. doi: 10.3389/fgene.2016.00137 (PMC4971393; doi:10.3389/fgene.2016.00137)
Supplement: Supplementary file 2 [file DataSheet2.pdf]

```
DATASET 1:
Mutation Calling Files:
provided by Patricia
TCGA_OV RNASeq downloaded from TCGA portal Dec 10, 2012
  [1] "unc.edu.ec1fc406-d1c4-4eba-ab6b-6cfb460f0862.1518127.rsem.genes.normalized_resu
lts"
  [2] "unc.edu.802ea3c6-e07f-49a1-a1c2-8f90e3197e62.1533373.rsem.genes.normalized_resu
lts"
  [3] "unc.edu.2535da6b-39f0-41de-afd3-82cb37916b95.1564423.rsem.genes.normalized_resu
lts"
  [4] "unc.edu.cc5e2498-a9e6-48be-bbda-9aa101546ca4.1520255.rsem.genes.normalized_resu
lts"
  [5] "unc.edu.32d5209e-2ef2-4ef0-b902-585542d7d16b.2723520.rsem.genes.normalized_resu
lts"
  [6] "unc.edu.529b7dd2-075c-4e93-b8c8-6990d3297c30.1549242.rsem.genes.normalized_resu
lts"
  [7] "unc.edu.a56ccd4c-1959-44a9-968d-57e6c5bc0cf8.1524214.rsem.genes.normalized_resu
lts"
  [8] "unc.edu.658b4847-33bc-414c-a2bb-ce9379ef57bd.1564763.rsem.genes.normalized_resu
lts"
  [9] "unc.edu.6bc296b8-ac74-4ff9-872d-5f7e4a3d011a.1550229.rsem.genes.normalized_resu
lts"
 [10] "unc.edu.754dfe07-22f4-4070-bbcb-10bfbb649def.1532261.rsem.genes.normalized_resu
lts"
 [11] "unc.edu.65cc3a15-cfa9-48fb-b9e4-801398b073b8.1580596.rsem.genes.normalized_resu
lts"
 [12] "unc.edu.479b74bf-5003-45e1-9505-687146a5fc45.1548619.rsem.genes.normalized_resu
lts"
 [13] "unc.edu.605d0aed-4637-4a09-82dd-eedd1aa76ca0.1565845.rsem.genes.normalized_resu
lts"
 [14] "unc.edu.63c52e46-68cf-4dac-9bd7-37081daa4474.1565124.rsem.genes.normalized_resu
lts"
 [15] "unc.edu.2d8e1b4d-85d0-4bfd-99f7-59559dabe978.1560256.rsem.genes.normalized_resu
lts"
 [16] "unc.edu.3217dd54-d41c-4c63-8bab-ff2c4baaee10.1564581.rsem.genes.normalized_resu
lts"
 [17] "unc.edu.044d949f-0eb4-44c9-8327-90226936fc7c.1552364.rsem.genes.normalized_resu
lts"
 [18] "unc.edu.fdda1428-ad6b-4337-a959-83bae1f9664b.1519083.rsem.genes.normalized_resu
lts"
 [19] "unc.edu.eb61bd52-e5fa-4c33-a2e4-1353a42184d9.1518550.rsem.genes.normalized_resu
lts"
 [20] "unc.edu.176d1ca2-eb1d-4f49-9b10-c129c822fa5a.1563786.rsem.genes.normalized_resu
lts"
 [21] "unc.edu.7774b7d4-2935-4b9b-bf76-312a625629e1.1532849.rsem.genes.normalized_resu
lts"
 [22] "unc.edu.41a5fa6b-4594-4ef8-b841-fe75c17a13c2.1555073.rsem.genes.normalized_resu
lts"
 [23] "unc.edu.edbc81a6-2593-45c5-8131-3c4615d1807f.1530426.rsem.genes.normalized_resu
lts"
 [24] "unc.edu.202abe19-20e0-4ea9-8653-bf5145da351c.1562761.rsem.genes.normalized_resu
lts"
 [25] "unc.edu.545867ef-1299-410d-bbbf-d6c189344b0f.1548271.rsem.genes.normalized_resu
lts"
 [26] "unc.edu.4e701e9f-9127-4d61-8a72-7e440b969fbe.1566440.rsem.genes.normalized_resu
lts"
 [27] "unc.edu.5a505632-422c-4083-b867-82a7a503b314.1565546.rsem.genes.normalized_resu
lts"
 [28] "unc.edu.368f21cd-dd07-440d-89f5-5874e37fe63b.2723112.rsem.genes.normalized_resu
lts"
 [29] "unc.edu.45ff767a-721a-4d89-b5e9-0c6e7eba476d.1564854.rsem.genes.normalized_resu
lts"
 [30] "unc.edu.4da3ccd5-53e1-46f5-874d-e5d131dbfc52.1555332.rsem.genes.normalized_resu
lts"
```

```
 [31] "unc.edu.f8ead0c8-9072-4a1d-9e07-f724a0d74a0f.1518741.rsem.genes.normalized_resu
lts"
 [32] "unc.edu.31e8ae73-7c3f-45ec-b4c9-d3f2ee1ca4fd.1564820.rsem.genes.normalized_resu
lts"
 [33] "unc.edu.bca48aa6-25bc-49e4-8bb5-edf38362b4a8.1520189.rsem.genes.normalized_resu
lts"
 [34] "unc.edu.eb8cb3b3-d1b1-4d2d-b048-7394931040bd.1519505.rsem.genes.normalized_resu
lts"
 [35] "unc.edu.03b8480f-d19e-43e4-8a09-f9230c525c1e.1552067.rsem.genes.normalized_resu
lts"
 [36] "unc.edu.f4187f4f-9c52-4065-818b-c884183a5a70.1519528.rsem.genes.normalized_resu
lts"
 [37] "unc.edu.a658045c-93a6-4aac-abbb-1cd6093b7735.1524641.rsem.genes.normalized_resu
lts"
 [38] "unc.edu.e78f43ff-0b31-4f9b-a9c1-02155ee3d8b0.1518878.rsem.genes.normalized_resu
lts"
 [39] "unc.edu.9ebb3535-6536-49fc-a88b-473235972378.1523469.rsem.genes.normalized_resu
lts"
 [40] "unc.edu.f3581696-e1db-4d0f-9ce7-5fb0f5c08906.1517237.rsem.genes.normalized_resu
lts"
 [41] "unc.edu.bdc4400c-c552-450b-a806-2ae10f61cca8.2722685.rsem.genes.normalized_resu
lts"
 [42] "unc.edu.7b39cb0a-7152-4037-9989-57545a1df980.1539636.rsem.genes.normalized_resu
lts"
 [43] "unc.edu.52697815-ede2-4ce3-a6d0-2680f6b9bb73.2722895.rsem.genes.normalized_resu
lts"
 [44] "unc.edu.790c6d79-9f7d-4e9a-89db-43b26fe16816.1536290.rsem.genes.normalized_resu
lts"
 [45] "unc.edu.5cf09ce6-44b1-4f0e-89ac-3ea302d684d8.1565016.rsem.genes.normalized_resu
lts"
 [46] "unc.edu.a3083fd8-f5d0-486b-bf3d-31ae094b1cbf.2722966.rsem.genes.normalized_resu
lts"
 [47] "unc.edu.29c4cab9-c19e-4416-ad88-484cc02a980f.1563461.rsem.genes.normalized_resu
lts"
 [48] "unc.edu.d10f0cee-d755-43cc-8d3d-8974cea0defb.1519303.rsem.genes.normalized_resu
lts"
 [49] "unc.edu.0ee0c708-77c4-4ac3-be13-0d08e6444003.1552261.rsem.genes.normalized_resu
lts"
 [50] "unc.edu.5c98c2c5-806f-4562-ad57-e152ea8388ab.1548784.rsem.genes.normalized_resu
lts"
 [51] "unc.edu.ab0701bc-0bc5-4008-a75b-5f665ef9c14a.1523606.rsem.genes.normalized_resu
lts"
 [52] "unc.edu.7b31fc47-0299-4449-aa5b-622ba549371f.1545332.rsem.genes.normalized_resu
lts"
 [53] "unc.edu.bb12d2f5-1865-435a-8edf-f9ee7689e0a4.2723310.rsem.genes.normalized_resu
lts"
 [54] "unc.edu.cd799c12-6a2b-4f7a-af72-f4bd7f69f6c7.1519895.rsem.genes.normalized_resu
lts"
 [55] "unc.edu.55b6bd7c-e77d-4019-93e9-b5410e4807b2.2722517.rsem.genes.normalized_resu
lts"
 [56] "unc.edu.9ec93bbd-5c62-4974-984f-f86301f0d27e.1526978.rsem.genes.normalized_resu
lts"
 [57] "unc.edu.3a37e320-0301-4f2b-bf09-f102c12502b1.1555655.rsem.genes.normalized_resu
lts"
 [58] "unc.edu.7e58cfd1-07eb-44e2-a563-fa7a00b338a3.1532645.rsem.genes.normalized_resu
lts"
 [59] "unc.edu.866c7355-e2ec-4816-953f-7bf791033283.1530482.rsem.genes.normalized_resu
lts"
 [60] "unc.edu.14cd154a-6ea5-4ad9-92e4-72f53ee5d60b.2723377.rsem.genes.normalized_resu
lts"
 [61] "unc.edu.10271f7f-cce6-44fd-8754-7a4beabfb394.1563533.rsem.genes.normalized_resu
lts"
 [62] "unc.edu.a391b870-b6fb-4ca8-b425-2331fceae442.1527198.rsem.genes.normalized_resu
lts"
```

```
 [63] "unc.edu.d76a99a6-18cd-4a50-9945-ebf5c5ccbfd1.1517257.rsem.genes.normalized_resu
lts"
 [64] "unc.edu.95970bdb-e1f2-49dc-903b-c30088cef71d.2723327.rsem.genes.normalized_resu
lts"
 [65] "unc.edu.c87ddfc3-d7a4-4704-b3cc-a19edfb63452.1519939.rsem.genes.normalized_resu
lts"
 [66] "unc.edu.1465139e-d1b2-4026-a532-08513fe0ad4d.1564255.rsem.genes.normalized_resu
lts"
 [67] "unc.edu.2fadaf5a-402b-483d-bc26-08feb3dc162d.1563088.rsem.genes.normalized_resu
lts"
 [68] "unc.edu.cfb1a6e0-f2d8-48bd-9303-fe7be5276ab7.1520306.rsem.genes.normalized_resu
lts"
 [69] "unc.edu.1f972766-2f58-4d41-b84c-3493ef2dd0c4.1563723.rsem.genes.normalized_resu
lts"
 [70] "unc.edu.759060d9-af2e-4098-b4ee-7aa2214acba8.1535813.rsem.genes.normalized_resu
lts"
 [71] "unc.edu.7b412fd9-4414-440f-b768-70666037c768.1530328.rsem.genes.normalized_resu
lts"
 [72] "unc.edu.9aa3ae15-1033-46f1-a304-993f5d41cd50.1524833.rsem.genes.normalized_resu
lts"
 [73] "unc.edu.cd93531b-4d92-4be3-95e5-032636dd1ad8.1518367.rsem.genes.normalized_resu
lts"
 [74] "unc.edu.2b2d5fc7-b109-4d11-a632-db8016268840.1563147.rsem.genes.normalized_resu
lts"
 [75] "unc.edu.cdaf98b1-fdb5-4575-aa17-e2d2ccba31f9.1520765.rsem.genes.normalized_resu
lts"
 [76] "unc.edu.85a4c41b-9829-48f8-9c7a-a9de870332b6.1527057.rsem.genes.normalized_resu
lts"
 [77] "unc.edu.0069c63d-f699-41d4-9798-9a109d1c09df.2723141.rsem.genes.normalized_resu
lts"
 [78] "unc.edu.761f9668-304f-4e22-87cd-2005aa86152c.1538714.rsem.genes.normalized_resu
lts"
 [79] "unc.edu.67e85198-4b8c-45db-b433-683b1899f3ea.1548669.rsem.genes.normalized_resu
lts"
 [80] "unc.edu.32de1e4d-198a-4616-8227-4d633c80124b.1555153.rsem.genes.normalized_resu
lts"
 [81] "unc.edu.1c88dde8-4fe6-49e9-8d76-40668ed783ff.2723481.rsem.genes.normalized_resu
lts"
 [82] "unc.edu.973907c7-f259-4d1a-b9df-68db1837af91.1525749.rsem.genes.normalized_resu
lts"
 [83] "unc.edu.9b64998e-eed9-428b-97ac-4de398caaf81.1526791.rsem.genes.normalized_resu
lts"
 [84] "unc.edu.54b75801-ad36-49a1-b0c2-edc0053b897e.1549219.rsem.genes.normalized_resu
lts"
 [85] "unc.edu.71543abc-9f15-48d9-994d-e7b963a4d8f3.1549380.rsem.genes.normalized_resu
lts"
 [86] "unc.edu.16c61c5a-a9b3-4022-9b79-d593e3519794.1563227.rsem.genes.normalized_resu
lts"
 [87] "unc.edu.4c8dd391-5a1c-490e-a7d7-fde372c3ec47.1572938.rsem.genes.normalized_resu
lts"
 [88] "unc.edu.67fcea16-d5bc-4edd-b66d-bfc5d56d976d.1548216.rsem.genes.normalized_resu
lts"
 [89] "unc.edu.e76a7745-3193-4650-9a7a-c96329690be3.1518580.rsem.genes.normalized_resu
lts"
 [90] "unc.edu.7bf43d51-2714-419e-a52f-863ca54c90e5.1533623.rsem.genes.normalized_resu
lts"
 [91] "unc.edu.d3fbcdaa-11af-4233-9700-57c6e1628c4b.1520042.rsem.genes.normalized_resu
lts"
 [92] "unc.edu.f580e84b-b405-43dc-9dae-4588cedf435f.2722918.rsem.genes.normalized_resu
lts"
 [93] "unc.edu.30b9b778-1422-4842-ae99-658b8b1d7605.1566225.rsem.genes.normalized_resu
lts"
 [94] "unc.edu.c20ba763-e310-448a-9c85-0b4f680e6196.2722663.rsem.genes.normalized_resu
lts"
```

```
 [95] "unc.edu.939d41b7-9ccb-4cf6-be16-f2792aece5eb.2723394.rsem.genes.normalized_resu
lts"
 [96] "unc.edu.7b6fe6e3-145b-46e6-884e-fcec23514674.1532957.rsem.genes.normalized_resu
lts"
 [97] "unc.edu.e2fcba09-755d-425d-ab4d-cede8ef86e48.2723451.rsem.genes.normalized_resu
lts"
 [98] "unc.edu.77b1c06b-46ef-4c8c-8a82-37f83e3c48cf.1535153.rsem.genes.normalized_resu
lts"
 [99] "unc.edu.0f5f479c-c83b-404b-bd17-ebb2aa5b0dc8.1551976.rsem.genes.normalized_resu
lts"
[100] "unc.edu.990bcb1f-7269-4020-8ea5-8ba338b390b8.1526603.rsem.genes.normalized_resu
lts"
[101] "unc.edu.7c607e77-a353-4995-855b-36ccaaf3bde6.1534254.rsem.genes.normalized_resu
lts"
[102] "unc.edu.0ec7ea28-7bd4-45c3-b03c-d855014fa4f4.1552652.rsem.genes.normalized_resu
lts"
[103] "unc.edu.1b8d5b5e-f671-419a-844a-6640fe5a896d.1563683.rsem.genes.normalized_resu
lts"
[104] "unc.edu.a7ea40ed-6cfb-42b7-92b7-64d6ba988414.1525061.rsem.genes.normalized_resu
lts"
[105] "unc.edu.a9c4a08c-1db7-49eb-8685-b5ca442c3e7a.1573921.rsem.genes.normalized_resu
lts"
[106] "unc.edu.97c0b20f-ee65-42b2-af2d-92dcf48b2cd5.1526854.rsem.genes.normalized_resu
lts"
[107] "unc.edu.c334b353-0652-49fb-8cd6-5bcf8485e5d0.1523401.rsem.genes.normalized_resu
lts"
[108] "unc.edu.52908652-f29a-4517-8ff6-6f4cd31c691b.1564933.rsem.genes.normalized_resu
lts"
[109] "unc.edu.bdd5afb1-a251-4541-bbbe-0fcd1126a3ce.1545652.rsem.genes.normalized_resu
lts"
[110] "unc.edu.e496319b-df71-44a0-bfa6-90f50ae54654.1517549.rsem.genes.normalized_resu
lts"
[111] "unc.edu.75e9506a-d007-4fb2-8bdd-7e7780653a63.1542199.rsem.genes.normalized_resu
lts"
[112] "unc.edu.efb87f4e-6fc7-4b2c-9d56-62b0f7834fbe.1517487.rsem.genes.normalized_resu
lts"
[113] "unc.edu.44948279-405d-42f6-ad29-465150ee6d85.1550120.rsem.genes.normalized_resu
lts"
[114] "unc.edu.dbc2cfac-df65-4750-af39-37760bad1057.1517871.rsem.genes.normalized_resu
lts"
[115] "unc.edu.a3173b33-aa16-48a2-b733-0345aad49755.1522780.rsem.genes.normalized_resu
lts"
[116] "unc.edu.ab99683f-6116-463d-8bf6-5782809d4729.1554719.rsem.genes.normalized_resu
lts"
[117] "unc.edu.b6908d33-80c8-4fc0-bd1d-829ec3161527.1523575.rsem.genes.normalized_resu
lts"
[118] "unc.edu.423f3c95-8bf5-4c2c-8b7a-343d9af1d0bf.1555270.rsem.genes.normalized_resu
lts"
[119] "unc.edu.4d3e0164-1346-4e8e-b478-29bd8b1f1ce5.1548596.rsem.genes.normalized_resu
lts"
[120] "unc.edu.8f0f34fb-bbef-4a17-8324-4ebda56f9e0e.1528464.rsem.genes.normalized_resu
lts"
[121] "unc.edu.74ad9d3e-97a9-419d-8842-3fb37d3f099a.1536188.rsem.genes.normalized_resu
lts"
[122] "unc.edu.bce04e1e-a90c-4e81-bd16-415cccc9d7e2.1524148.rsem.genes.normalized_resu
lts"
[123] "unc.edu.b29bd766-95b1-46fa-a070-d2206cd5722a.1526907.rsem.genes.normalized_resu
lts"
[124] "unc.edu.3e4f0ca7-8428-47df-80a3-3bc9153aedfa.1566392.rsem.genes.normalized_resu
lts"
[125] "unc.edu.921fab94-5522-4c08-8e02-13fb6ea2d388.1533658.rsem.genes.normalized_resu
lts"
[126] "unc.edu.daaba89d-acd3-4670-a618-89e5973b03f4.2722778.rsem.genes.normalized_resu
lts"
```

[127] "unc.edu.0fc68715-c7fc-4481-a5bf-68a281f5ec64.1563553.rsem.genes.normalized_resu
lts"
[128] "unc.edu.c5a82b6e-048d-4377-9b6c-4417ba1876fe.1520117.rsem.genes.normalized_resu
lts"
[129] "unc.edu.12c78d62-6045-4f77-bd84-9ab5a38f4635.1563628.rsem.genes.normalized_resu
lts"
[130] "unc.edu.7517c446-401d-4501-bf27-916e0835deac.1540345.rsem.genes.normalized_resu
lts"
[131] "unc.edu.877d96a2-6e5c-472c-b190-59fcfd1febb4.1542858.rsem.genes.normalized_resu
lts"
[132] "unc.edu.95b7a71d-8cf5-4660-ac1d-52a888cd2f20.1555182.rsem.genes.normalized_resu
lts"
[133] "unc.edu.43352a4d-f866-4ed7-81f5-5dc478c10992.1549837.rsem.genes.normalized_resu
lts"
[134] "unc.edu.28a681dc-3e0f-4a9c-ad0c-f2bb10aab0e1.1565980.rsem.genes.normalized_resu
lts"
[135] "unc.edu.c3a1cbaf-28cd-451c-ad93-5a03a61a8a2c.2723530.rsem.genes.normalized_resu
lts"
[136] "unc.edu.05ab6bb9-0cb1-47f1-8dd2-92a48239e82e.1552874.rsem.genes.normalized_resu
lts"
[137] "unc.edu.4e5f3a15-68fd-4be0-b368-e0bd04eaf872.1550168.rsem.genes.normalized_resu
lts"
[138] "unc.edu.f1579d6e-6efd-47b5-b2b4-a97e1480ce0a.1517888.rsem.genes.normalized_resu
lts"
[139] "unc.edu.690d1437-2a05-4263-8fa7-8f4481893b83.1549161.rsem.genes.normalized_resu
lts"
[140] "unc.edu.a32eba80-dbe9-464e-8102-5f77a3c7e177.1524859.rsem.genes.normalized_resu
lts"
[141] "unc.edu.25b1eb9f-7b97-4da2-96a9-a4a3aaaca192.2722216.rsem.genes.normalized_resu
lts"
[142] "unc.edu.4e539c62-454a-4b1c-bde1-6951ee12d109.1550257.rsem.genes.normalized_resu
lts"
[143] "unc.edu.55c68aef-938c-4f68-a74d-f408673fff9c.1550239.rsem.genes.normalized_resu
lts"
[144] "unc.edu.ff781a4f-7281-4195-880f-3df099a401cc.1519264.rsem.genes.normalized_resu
lts"
[145] "unc.edu.a28ef775-c78b-416f-afeb-89ba265440f0.1527412.rsem.genes.normalized_resu
lts"
[146] "unc.edu.7ecb58fe-e2fe-4dc8-996b-2b959da43566.1535519.rsem.genes.normalized_resu
lts"
[147] "unc.edu.6a101b07-3cc5-4da3-90ab-0e45fa5b9cd3.1565256.rsem.genes.normalized_resu
lts"
[148] "unc.edu.cb7a4405-b9a5-4e1a-825f-c3ee205c8a90.1520212.rsem.genes.normalized_resu
lts"
[149] "unc.edu.e92b95df-bacb-4083-b2de-78e540ec0e68.1518096.rsem.genes.normalized_resu
lts"
[150] "unc.edu.55f9061c-54e7-465c-af61-21d8bc8ecbe0.1548322.rsem.genes.normalized_resu
lts"
[151] "unc.edu.7162d809-ff1a-4346-bb00-c8086386ec46.1534136.rsem.genes.normalized_resu
lts"
[152] "unc.edu.e05f2e2a-77d2-4f86-8e21-27cdd93b88b0.1518533.rsem.genes.normalized_resu
lts"
[153] "unc.edu.a209a8f4-9462-4ade-be57-caaec54999cb.1522713.rsem.genes.normalized_resu
lts"
[154] "unc.edu.33367eb6-4ca9-4daf-9e47-30a47a78e144.2723352.rsem.genes.normalized_resu
lts"
[155] "unc.edu.1d3f29ac-5f19-452d-99b6-72c0abf1ce32.1563056.rsem.genes.normalized_resu
lts"
[156] "unc.edu.bf909020-d6a1-49f1-8007-4192d39abd04.1526065.rsem.genes.normalized_resu
lts"
[157] "unc.edu.89f1ae39-c387-4dff-be38-e3b7265222bb.1528824.rsem.genes.normalized_resu
lts"
[158] "unc.edu.056cca38-92df-42ff-b064-b0c243f8a82f.1552149.rsem.genes.normalized_resu
lts"

[159] "unc.edu.13b2c972-c6ef-4d1f-8ada-f831d2606023.1563882.rsem.genes.normalized_results"
[160] "unc.edu.0814c20e-fe77-4ff9-ab5e-a8ae2f74069d.1551746.rsem.genes.normalized_results"
[161] "unc.edu.f4fccaa6-cd8c-4e5a-a7a8-beb63cf7b109.1518791.rsem.genes.normalized_results"
[162] "unc.edu.bc200f3a-6d31-4480-8785-987f14cfa319.2722841.rsem.genes.normalized_results"
[163] "unc.edu.b6ed2184-0885-49ba-8b00-a3e57e4e5500.1524467.rsem.genes.normalized_results"
[164] "unc.edu.929c2bc4-4cee-4f82-b336-918fe041a588.1526644.rsem.genes.normalized_results"
[165] "unc.edu.0ed6e7a9-cdaa-48dc-962f-219f1eb39c1a.1552050.rsem.genes.normalized_results"
[166] "unc.edu.c6e1c591-3225-482a-8350-112b664a37e6.1564827.rsem.genes.normalized_results"
[167] "unc.edu.7ac6af79-c14a-4026-84aa-a288afcb96da.1530431.rsem.genes.normalized_results"
[168] "unc.edu.721a8fc8-bb54-4ec3-a19c-340c35f05280.1533564.rsem.genes.normalized_results"
[169] "unc.edu.6561cdb8-8499-41a6-8a79-ef0119e34c66.1548926.rsem.genes.normalized_results"
[170] "unc.edu.25c0888a-25b9-4cc0-a02f-6d363a341e8f.1560278.rsem.genes.normalized_results"
[171] "unc.edu.d5752594-1a2c-47c8-a40b-bffd5b6b7e71.1525735.rsem.genes.normalized_results"

DATASET 2:
Mutation Calling Files:
genome.wustl.edu_BRCA.IlluminaGA_DNASeq.Level_2.5.3.0.somatic.maf
genome.wustl.edu_BRCA.IlluminaGA_DNASeq.Level_2.1.1.0.curated.somatic.maf

TCGA_BRCA RNASeq downloaded from TCGA portal Jan 28, 2015"
[1] "unc.edu.6d066a72-f59f-45a8-ab90-216000b36da4.1132519.rsem.genes.normalized_results"
[2] "unc.edu.6e45dd01-225d-4871-9a3a-f591808ad234.1133882.rsem.genes.normalized_results"
[3] "unc.edu.5f5a884c-ffbd-401a-8689-baded1bdbe50.1289271.rsem.genes.normalized_results"
[4] "unc.edu.42e2160c-07b5-4be6-a989-1e3474926d2a.1123062.rsem.genes.normalized_results"
[5] "unc.edu.c0dd6c33-008d-44e8-b20c-8089bc3e29a3.1132984.rsem.genes.normalized_results"
[6] "unc.edu.a74b20ca-06f9-47ec-8888-cb01e0ba3257.1121362.rsem.genes.normalized_results"
[7] "unc.edu.b4673313-fa18-43e8-a01b-5cdb3cf5425e.1127416.rsem.genes.normalized_results"
[8] "unc.edu.ec38c31b-def8-41cc-8656-57ce98f3faa7.1119113.rsem.genes.normalized_results"
[9] "unc.edu.91a5b906-6a04-4f0d-8710-172311e84789.1121069.rsem.genes.normalized_results"
[10] "unc.edu.50674dde-30c1-4530-a164-381d5326e29c.1171265.rsem.genes.normalized_results"
[11] "unc.edu.66f3892f-f4ec-412c-9c56-a9e9b56a15ee.1117815.rsem.genes.normalized_results"
[12] "unc.edu.0c7fc003-950b-4138-8390-036c072b640e.1118684.rsem.genes.normalized_results"
[13] "unc.edu.39413018-090f-46ca-b6df-244124f098c3.1102960.rsem.genes.normalized_results"
[14] "unc.edu.81239059-614b-46b7-a21e-224ec62e2939.1106224.rsem.genes.normalized_results"
[15] "unc.edu.8cf1853a-55b2-4fb9-9a65-a46243304d54.1162825.rsem.genes.normalized_results"
[16] "unc.edu.60534f8f-970a-4308-9844-a3a41dd6c187.1164550.rsem.genes.normalized_resul

```
ts"
[17] "unc.edu.fb03a31f-6611-4343-816d-37a332147eac.1132304.rsem.genes.normalized_resul
ts"
[18] "unc.edu.5213da80-35ac-4172-b2e8-de686e73a498.1129191.rsem.genes.normalized_resul
ts"
[19] "unc.edu.0a69935d-9552-4792-9b2e-1a630114691e.1127132.rsem.genes.normalized_resul
ts"
[20] "unc.edu.123725b8-4f66-4b97-9726-e0c70300d38e.1119933.rsem.genes.normalized_resul
ts"
[21] "unc.edu.20486352-7d5f-4065-ab10-7a3f792ec1e1.1119295.rsem.genes.normalized_resul
ts"
[22] "unc.edu.83f8c292-5ae0-4c58-9f08-e58ce391f4f4.1286013.rsem.genes.normalized_resul
ts"
[23] "unc.edu.ec2ac902-f5ca-45a5-a72a-1b266360410a.1283013.rsem.genes.normalized_resul
ts"
[24] "unc.edu.d848ad65-9261-4854-a6bd-e76484c6a337.1261526.rsem.genes.normalized_resul
ts"
[25] "unc.edu.9f693816-6957-413c-a100-54f7bc0eddf9.1265196.rsem.genes.normalized_resul
ts"
[26] "unc.edu.e5aeb7a3-1454-4605-a924-d4b1ed082e87.1265224.rsem.genes.normalized_resul
ts"
[27] "unc.edu.b5b8fdd8-c4df-4f79-aed5-23ac41f75f18.1349498.rsem.genes.normalized_resul
ts"
[28] "unc.edu.4763b4dd-1eef-4503-9381-4baa57cc690f.1929036.rsem.genes.normalized_resul
ts"
[29] "unc.edu.e0a10445-6266-4f41-a747-fb19d7182652.1807220.rsem.genes.normalized_resul
ts"
[30] "unc.edu.043d5dcd-1524-402b-939c-984a1dc916a0.1806901.rsem.genes.normalized_resul
ts"
[31] "unc.edu.42c88ed3-de96-487d-8b03-e1d4269d0505.1807503.rsem.genes.normalized_resul
ts"
[32] "unc.edu.976a1b88-ed7a-493a-8ac9-3d504edfbc76.1806314.rsem.genes.normalized_resul
ts"
[33] "unc.edu.6bb217e7-3fd4-477f-87bf-b55e50a33e45.1989125.rsem.genes.normalized_resul
ts"
[34] "unc.edu.7810122d-fa8b-483d-9398-aa26fc7fedc5.2028954.rsem.genes.normalized_resul
ts"
[35] "unc.edu.16f90247-c9a5-40d5-a3a1-08bba2d1d266.2090212.rsem.genes.normalized_resul
ts"
[36] "unc.edu.4b5b44ea-8589-4aea-a69f-b5d5041b1acd.2032690.rsem.genes.normalized_resul
ts"
[37] "unc.edu.bd51f90a-ec67-4a7d-87bf-9d85acd65d51.1173496.rsem.genes.normalized_resul
ts"
[38] "unc.edu.0b743ba9-a340-46a0-9276-a5ddd3ee69d6.1114431.rsem.genes.normalized_resul
ts"
[39] "unc.edu.f97f68be-6838-455c-91bf-7549543a82c3.1159138.rsem.genes.normalized_resul
ts"
[40] "unc.edu.467314d6-f946-447f-920a-d33b60f66eb2.1185391.rsem.genes.normalized_resul
ts"
[41] "unc.edu.12c36b7c-f302-4cea-bb4f-5a01211e00e9.2196009.rsem.genes.normalized_resul
ts"
[42] "unc.edu.62dd11c2-2271-45e7-b753-d0f0d79fdf23.1157441.rsem.genes.normalized_resul
ts"
[43] "unc.edu.2ad8d416-08a4-4e6f-947e-d74269d02ab1.1154057.rsem.genes.normalized_resul
ts"
[44] "unc.edu.19d713e8-e754-44a4-9b23-0ef70f5d6b44.1162794.rsem.genes.normalized_resul
ts"
[45] "unc.edu.3a1d8959-f829-4b8a-a65f-70a51845f118.1162769.rsem.genes.normalized_resul
ts"
[46] "unc.edu.c09e8a88-5a82-4fd6-8d03-ef3ecb988003.1148322.rsem.genes.normalized_resul
ts"
[47] "unc.edu.4808bc63-000a-4a49-a25b-4b817ca5ea54.1150371.rsem.genes.normalized_resul
ts"
[48] "unc.edu.1fe98b05-6803-4803-83cd-a59e794b956e.1148441.rsem.genes.normalized_resul
```

```
ts"
[49] "unc.edu.97168a1f-abf8-414b-af10-b63f5daa7023.1174256.rsem.genes.normalized_resul
ts"
[50] "unc.edu.7953e62d-4871-4928-a2ba-a38c740134e1.1153547.rsem.genes.normalized_resul
ts"
[51] "unc.edu.6d2744ba-4817-482b-ad46-2e4e0897ad88.1153745.rsem.genes.normalized_resul
ts"
[52] "unc.edu.d5c39754-79c3-432c-b496-34a394662e17.1675649.rsem.genes.normalized_resul
ts"
[53] "unc.edu.fac9101c-a974-4415-9eac-d2d433d02b13.1149728.rsem.genes.normalized_resul
ts"
[54] "unc.edu.a63583ca-0ef2-4417-b435-5f3a6951d0c2.1153349.rsem.genes.normalized_resul
ts"
[55] "unc.edu.f091d050-4847-4d4d-b94c-1efacecdef6b.1152654.rsem.genes.normalized_resul
ts"
[56] "unc.edu.28f9b0d6-8b8f-4dd6-8a7d-73cdd374a825.1152749.rsem.genes.normalized_resul
ts"
[57] "unc.edu.491c6c41-d1c5-4be7-bb7c-9df7275aa388.1153803.rsem.genes.normalized_resul
ts"
[58] "unc.edu.5c911cb7-4e7b-4ffd-bd2b-4498ee7b0e88.1152638.rsem.genes.normalized_resul
ts"
[59] "unc.edu.7f57b9e7-1b1b-4e07-beb4-b877b7651f7b.1143257.rsem.genes.normalized_resul
ts"
[60] "unc.edu.74cba80b-7677-41d6-ab22-10f1a962ba2f.1171722.rsem.genes.normalized_resul
ts"
[61] "unc.edu.ee7156b6-f469-402c-8f63-f4c2f6913db1.1144705.rsem.genes.normalized_resul
ts"
[62] "unc.edu.519e1ea1-6658-4978-9f3d-313d7cc1e0c1.1114987.rsem.genes.normalized_resul
ts"
[63] "unc.edu.5cd42d4a-5971-4a01-8c10-99321105a864.1145544.rsem.genes.normalized_resul
ts"
[64] "unc.edu.4a93eaee-d821-4640-a441-255008aaf3b4.1145090.rsem.genes.normalized_resul
ts"
[65] "unc.edu.15b9736e-eafc-465e-9ddd-b1b498e8b701.1145675.rsem.genes.normalized_resul
ts"
[66] "unc.edu.4d12d021-3673-4e14-9fd9-033f811f87da.1142876.rsem.genes.normalized_resul
ts"
[67] "unc.edu.9d04c180-7c23-490e-92cf-c018629b8b7f.1136428.rsem.genes.normalized_resul
ts"
[68] "unc.edu.3e57b40c-7079-4f05-bf37-f560304966c1.1134101.rsem.genes.normalized_resul
ts"
[69] "unc.edu.70b29dd6-1688-4510-94dd-5fcb26a1a908.1141734.rsem.genes.normalized_resul
ts"
[70] "unc.edu.39ca8590-3733-4b61-9a46-4ec5e6e0501e.1136610.rsem.genes.normalized_resul
ts"
[71] "unc.edu.5162ef6d-6978-4ff7-b385-a59325cefe17.1134237.rsem.genes.normalized_resul
ts"
[72] "unc.edu.624ac458-8ff1-441c-a8c5-aaf4a08a4b84.1134256.rsem.genes.normalized_resul
ts"
[73] "unc.edu.4bf5f56d-448a-426a-8edc-f1bdbf6a8d55.1142205.rsem.genes.normalized_resul
ts"
[74] "unc.edu.4d28bcbc-dbdd-46f8-9329-49f359480a97.1142080.rsem.genes.normalized_resul
ts"
[75] "unc.edu.76a625e9-22b3-42b8-8813-27c133d0b248.1134459.rsem.genes.normalized_resul
ts"
[76] "unc.edu.d9f827bb-2498-49b0-aa85-71cb554eb926.1147661.rsem.genes.normalized_resul
ts"
[77] "unc.edu.429b50eb-316f-459c-bc3a-0aca6e6dba46.1131392.rsem.genes.normalized_resul
ts"
[78] "unc.edu.46262a6c-8a11-4bb9-8851-8b6e44c95288.1130625.rsem.genes.normalized_resul
ts"
[79] "unc.edu.43326f71-c61c-410b-af4b-c71a054e27c9.1130962.rsem.genes.normalized_resul
ts"
[80] "unc.edu.49c4166c-701a-4f53-8968-99aec3347214.1132885.rsem.genes.normalized_resul
```

ts"

DATASET3:
Mutation Files:
PR_TCGA_LUAD_PAIR_Capture_All_Pairs_QCPASS_v3.aggregated.capture.tcga.uuid.somatic.maf
PR_TCGA_LUAD_PAIR_Capture_All_Pairs_QCPASS_v4.aggregated.capture.tcga.uuid.automated.somatic.maf

TCGA_LUAD RNASeq downloaded from TCGA portal Jan 28, 2015
[1] "unc.edu.e4177b01-6898-4bb7-b38d-0c09f85c5668.1469055.rsem.genes.normalized_results"
[2] "unc.edu.7d6cf896-b04a-431a-a192-aaf540eeaf77.1466756.rsem.genes.normalized_results"
[3] "unc.edu.3ae6f6d4-ae90-4572-9bd5-ed8700570b96.1468237.rsem.genes.normalized_results"
[4] "unc.edu.82b154a5-b4d5-4737-8f79-10d252741963.1227721.rsem.genes.normalized_results"
[5] "unc.edu.e8fb125c-3249-44f4-9bc7-5cd086011d89.1221267.rsem.genes.normalized_results"
[6] "unc.edu.8835ae28-831e-49da-a046-a1a43b626a4e.1221387.rsem.genes.normalized_results"
[7] "unc.edu.86973b83-4077-414a-ad8d-dbf32b6707d1.1221251.rsem.genes.normalized_results"
[8] "unc.edu.a1510fc9-aff1-4cc2-8d8e-9aafce9319f7.1227826.rsem.genes.normalized_results"
[9] "unc.edu.149e2d5d-3d6c-410e-b435-7e37b3f96661.1227114.rsem.genes.normalized_results"
[10] "unc.edu.7f9f381e-edb1-4a5e-b5f7-c31b2a769912.1228449.rsem.genes.normalized_results"
[11] "unc.edu.89dda3b6-2905-4577-9b74-965fb7f77c98.1112349.rsem.genes.normalized_results"
[12] "unc.edu.c4da944d-4055-493e-bb87-7ccd1e5d1a87.1111523.rsem.genes.normalized_results"
[13] "unc.edu.b2aba2a6-3b30-41bb-a4cc-74f36b60a114.1112819.rsem.genes.normalized_results"
[14] "unc.edu.fd4a3155-ca05-464c-b8d9-816103de489d.1110998.rsem.genes.normalized_results"
[15] "unc.edu.bda96534-9900-4f6f-ab17-a09f52dfada4.1115497.rsem.genes.normalized_results"
[16] "unc.edu.31769d4d-64f5-4a2b-b8da-d5b2252badb3.1117546.rsem.genes.normalized_results"
[17] "unc.edu.013f7a4f-eec9-4ba8-8473-6515cdc9be68.1210300.rsem.genes.normalized_results"
[18] "unc.edu.44d80508-53d8-4ba3-b263-8c31e4b58096.1210248.rsem.genes.normalized_results"
[19] "unc.edu.46b786be-8c30-4025-aa29-708c76bb4bcf.1104036.rsem.genes.normalized_results"
[20] "unc.edu.7dfdb65a-8f75-4223-8e5c-bb13f1d8eb3b.1102054.rsem.genes.normalized_results"
[21] "unc.edu.9d983613-1588-48a2-a515-7afae7aafc92.1213954.rsem.genes.normalized_results"
[22] "unc.edu.2ef846b1-a0f9-412e-a72e-6dab6543d41f.1211890.rsem.genes.normalized_results"
[23] "unc.edu.ab215c47-4678-4ca9-8cd3-ac6e3e804c05.1204084.rsem.genes.normalized_results"
[24] "unc.edu.bc6c762f-727f-43c8-a4bc-4c41351c7f13.1503055.rsem.genes.normalized_results"
[25] "unc.edu.6ed236e4-faf1-4d23-bccd-341688b034c3.1229750.rsem.genes.normalized_results"
[26] "unc.edu.09a88dca-7503-4fcb-b42a-ac5af9fdbca7.1230118.rsem.genes.normalized_results"
[27] "unc.edu.b3cb327b-b0d4-478f-8dac-75abdb6365eb.1466630.rsem.genes.normalized_results"
[28] "unc.edu.b73f5912-d163-4812-995c-0a273f543af1.1466296.rsem.genes.normalized_resu

```
lts"
 [29] "unc.edu.7b9dc578-8869-4322-a705-bfb9942d9b65.1114490.rsem.genes.normalized_resu
lts"
 [30] "unc.edu.6f748364-2a6a-4fd4-af87-3de643719577.1114110.rsem.genes.normalized_resu
lts"
 [31] "unc.edu.5ec48286-3f47-49eb-83ba-a1169f734bba.1099164.rsem.genes.normalized_resu
lts"
 [32] "unc.edu.bfad522f-6130-424d-8497-99cac9714966.1212962.rsem.genes.normalized_resu
lts"
 [33] "unc.edu.4c5c1c05-b9d1-4073-9d91-5c4e552928ae.1105320.rsem.genes.normalized_resu
lts"
 [34] "unc.edu.8b0a1ab6-e7ea-45f9-9e3a-bd98b3f59972.1230273.rsem.genes.normalized_resu
lts"
 [35] "unc.edu.d9f5e680-b72e-4a64-942b-191154d8012a.1213431.rsem.genes.normalized_resu
lts"
 [36] "unc.edu.a8ef928f-7321-4355-b136-cec50ebd9481.1112210.rsem.genes.normalized_resu
lts"
 [37] "unc.edu.44205bf0-b593-4c0d-9aeb-4e604314e10f.1205865.rsem.genes.normalized_resu
lts"
 [38] "unc.edu.b4bbc93a-5460-476d-a663-61299fddfab2.1110187.rsem.genes.normalized_resu
lts"
 [39] "unc.edu.2d452408-9d25-49e1-81db-542021eb28cb.1466649.rsem.genes.normalized_resu
lts"
 [40] "unc.edu.29af0307-5d76-462d-beee-18e8c4f179b8.1210542.rsem.genes.normalized_resu
lts"
 [41] "unc.edu.7d27cfbf-870f-45e8-80a9-7e3848f414dc.1227546.rsem.genes.normalized_resu
lts"
 [42] "unc.edu.6a4b7295-a738-4d4a-a264-72fba462e60c.1110942.rsem.genes.normalized_resu
lts"
 [43] "unc.edu.25f0eee1-8f38-4e75-accf-7e7bb686405c.1110135.rsem.genes.normalized_resu
lts"
 [44] "unc.edu.ee90d7cd-4ac9-4be0-b9a8-49329890f69d.1110545.rsem.genes.normalized_resu
lts"
 [45] "unc.edu.57f72fed-dcb9-4bba-8810-30c254f4635c.1206656.rsem.genes.normalized_resu
lts"
 [46] "unc.edu.bbf1f651-44f0-42c9-a519-64f3d47b42dc.1443924.rsem.genes.normalized_resu
lts"
 [47] "unc.edu.d037d7f2-3277-4dc3-954b-503f906bc467.1438435.rsem.genes.normalized_resu
lts"
 [48] "unc.edu.0216208c-fbbd-4370-93dd-19fbb7703568.1441733.rsem.genes.normalized_resu
lts"
 [49] "unc.edu.7dcc9d07-9af9-405e-b75a-c0e7ffdff5c0.1438605.rsem.genes.normalized_resu
lts"
 [50] "unc.edu.109f2b77-1742-4afa-bcba-b763746fe080.1439260.rsem.genes.normalized_resu
lts"
 [51] "unc.edu.0b8ef0be-c6fb-4ca9-9da0-f97dd22767bf.1440643.rsem.genes.normalized_resu
lts"
 [52] "unc.edu.4bd54818-dbda-44f7-837e-0235716712d9.1327295.rsem.genes.normalized_resu
lts"
 [53] "unc.edu.8af91e43-8345-432a-a758-1ce8aa55c664.1325661.rsem.genes.normalized_resu
lts"
 [54] "unc.edu.9de618ee-04ff-49da-830a-f82eff981616.1966447.rsem.genes.normalized_resu
lts"
 [55] "unc.edu.64f41383-d668-489a-9fe7-f00235015442.1966594.rsem.genes.normalized_resu
lts"
 [56] "unc.edu.89b3869c-aacb-4882-8aa0-4d9058cf5625.1991130.rsem.genes.normalized_resu
lts"
 [57] "unc.edu.5f83fb8f-01d8-4deb-89d1-2fe9fb69255e.1991708.rsem.genes.normalized_resu
lts"
 [58] "unc.edu.3cc3fe41-c7e8-4dcf-99a0-a488f7763e93.1209947.rsem.genes.normalized_resu
lts"
 [59] "unc.edu.94a591be-8b32-4680-b2ad-fc856f3f0c8e.1102450.rsem.genes.normalized_resu
lts"
 [60] "unc.edu.5e45c9ab-f5fe-497b-b919-72b0d8dec83d.1209600.rsem.genes.normalized_resu
```

```
lts"
 [61] "unc.edu.cb06be5e-7c1d-49a4-9818-e1cbc52de9b6.1121209.rsem.genes.normalized_resu
lts"
 [62] "unc.edu.4b05bcbb-08b4-4cf5-bd31-798490e00f94.1109801.rsem.genes.normalized_resu
lts"
 [63] "unc.edu.6a1b9b24-7377-4807-a876-282f9d948473.1107725.rsem.genes.normalized_resu
lts"
 [64] "unc.edu.7a4e94fa-09fb-4483-8816-f3ff2f1b2a6f.1889028.rsem.genes.normalized_resu
lts"
 [65] "unc.edu.299d2ad8-b54d-4ddb-b6c5-b80d8baf39f2.1112263.rsem.genes.normalized_resu
lts"
 [66] "unc.edu.554a301f-fbc8-41f2-96e9-5da2ee943727.1228549.rsem.genes.normalized_resu
lts"
 [67] "unc.edu.10c6a96f-b119-43a8-8dfa-9af13bce53b9.1227797.rsem.genes.normalized_resu
lts"
 [68] "unc.edu.ea55c131-97f8-49cc-a445-87523ce1f075.1208749.rsem.genes.normalized_resu
lts"
 [69] "unc.edu.e2a7336c-5262-433a-ad52-8834eaee2e1e.1210478.rsem.genes.normalized_resu
lts"
 [70] "unc.edu.51eae0de-8f16-4093-b42e-5c34c4768459.1085524.rsem.genes.normalized_resu
lts"
 [71] "unc.edu.3cafb1e5-9de0-4ced-8b87-d2299a207b97.1227510.rsem.genes.normalized_resu
lts"
 [72] "unc.edu.84138ee7-b120-4201-95b1-ccdc1c82ebda.1098784.rsem.genes.normalized_resu
lts"
 [73] "unc.edu.c1859475-12fd-47f6-887d-136d334399cd.1110473.rsem.genes.normalized_resu
lts"
 [74] "unc.edu.8a5cfa5b-2dab-4775-ae08-3d7e0b36e93f.1087480.rsem.genes.normalized_resu
lts"
 [75] "unc.edu.12fdf681-a6f5-4dfe-b556-d7e7f7e4d58a.1111869.rsem.genes.normalized_resu
lts"
 [76] "unc.edu.92321ab5-8c77-4208-9daf-e6fcb01cc7a1.1111046.rsem.genes.normalized_resu
lts"
 [77] "unc.edu.444662df-ae9b-4fec-9afc-df392ba3a88d.1113309.rsem.genes.normalized_resu
lts"
 [78] "unc.edu.ace0aace-27ba-42a6-8d6b-3c29095ccbe5.1110082.rsem.genes.normalized_resu
lts"
 [79] "unc.edu.7326b41e-46e0-47fb-895d-43ef08c91b97.1109233.rsem.genes.normalized_resu
lts"
 [80] "unc.edu.cb72fb22-d492-432c-8188-82fce70c071c.1104102.rsem.genes.normalized_resu
lts"
 [81] "unc.edu.5655a240-2ed7-469e-af17-9997547ea119.1102692.rsem.genes.normalized_resu
lts"
 [82] "unc.edu.42a4a133-ec9d-4f5a-8b95-be83cbddb148.1943376.rsem.genes.normalized_resu
lts"
 [83] "unc.edu.50d03646-1082-4ae3-9853-f5295006f11a.1434320.rsem.genes.normalized_resu
lts"
 [84] "unc.edu.f3196cf4-2cda-4377-9089-e29434cbd4d4.1441102.rsem.genes.normalized_resu
lts"
 [85] "unc.edu.465ce132-c1f4-490f-b1f1-46300fde4441.1991677.rsem.genes.normalized_resu
lts"
 [86] "unc.edu.f215665e-3c8d-4746-a18a-859ac95b2fdb.1105957.rsem.genes.normalized_resu
lts"
 [87] "unc.edu.63b6924e-921f-4fac-9866-dd97e996d7ac.1103459.rsem.genes.normalized_resu
lts"
 [88] "unc.edu.8b2f9a82-4c0d-496b-a46c-15d20d069e11.1108596.rsem.genes.normalized_resu
lts"
 [89] "unc.edu.f51ff2e2-bc0a-4cdd-b008-933a8f7fa8fc.1227241.rsem.genes.normalized_resu
lts"
 [90] "unc.edu.f5a01f2f-247a-42a0-a713-ba6f8bb8d03e.1208652.rsem.genes.normalized_resu
lts"
 [91] "unc.edu.09fba9fa-29d2-41a1-b3aa-4bfd97be3fc8.1102310.rsem.genes.normalized_resu
lts"
 [92] "unc.edu.a4c1e9e8-a335-47b0-afc6-c2b490da4e58.1103042.rsem.genes.normalized_resu
```

```
lts"
 [93] "unc.edu.7ee4963f-9682-46b1-8946-4c726fd81c39.1203658.rsem.genes.normalized_resu
lts"
 [94] "unc.edu.a7a6d133-a7d0-44ea-bac5-3a8b7ddd9cd8.1102585.rsem.genes.normalized_resu
lts"
 [95] "unc.edu.fc852193-d692-4a36-83bd-30f49df5f973.1102369.rsem.genes.normalized_resu
lts"
 [96] "unc.edu.987c7eea-648f-4feb-8239-0b9151a74331.1210709.rsem.genes.normalized_resu
lts"
 [97] "unc.edu.91449ec2-a03f-4cc8-8738-4819564d9dae.1094515.rsem.genes.normalized_resu
lts"
 [98] "unc.edu.56982118-0f10-47b2-af05-18b037c73f22.1099474.rsem.genes.normalized_resu
lts"
 [99] "unc.edu.f9faf081-c497-44a3-9df1-da2be95e41d0.1204560.rsem.genes.normalized_resu
lts"
[100] "unc.edu.bbf4c600-a4c5-4ad6-8842-d90639aab690.1207863.rsem.genes.normalized_resu
lts"
[101] "unc.edu.829b31ba-7ab2-475e-bad9-1d1a9b6ab13f.1443782.rsem.genes.normalized_resu
lts"
[102] "unc.edu.4be7594c-c6ff-47c3-bb57-22ba6489b0f4.1084985.rsem.genes.normalized_resu
lts"
[103] "unc.edu.b2d849d8-f837-43e4-b06c-051f041172ae.1085035.rsem.genes.normalized_resu
lts"
[104] "unc.edu.d4349207-30b1-44ad-b460-14db8ea58cc4.1085197.rsem.genes.normalized_resu
lts"
[105] "unc.edu.514e90fa-113f-48d7-9166-41096e0ad209.1080070.rsem.genes.normalized_resu
lts"
[106] "unc.edu.0168f22b-6d11-4492-9aea-5ffa451bff3e.1078920.rsem.genes.normalized_resu
lts"
[107] "unc.edu.4ff760c4-074e-4876-b63f-71098942c780.1079342.rsem.genes.normalized_resu
lts"
[108] "unc.edu.9142e848-c5cb-41c1-b80f-5f7674f9a1cb.1080270.rsem.genes.normalized_resu
lts"
[109] "unc.edu.5bfe179e-eae2-4d2a-9f2a-b4af4602a2b5.1080016.rsem.genes.normalized_resu
lts"
[110] "unc.edu.c857b341-4021-48e6-a9e7-fff0525cdb96.1382672.rsem.genes.normalized_resu
lts"
[111] "unc.edu.956590e9-4962-497b-a59f-81ee0a1c0caf.1384677.rsem.genes.normalized_resu
lts"
[112] "unc.edu.04104d50-dcf7-4e77-9038-8fed73db6a12.1326394.rsem.genes.normalized_resu
lts"
[113] "unc.edu.671a4dd5-cb2a-4d9b-aec7-c5963d50f2ab.1326831.rsem.genes.normalized_resu
lts"
[114] "unc.edu.add23090-21e1-4cc2-a833-539b1db1e05f.1328639.rsem.genes.normalized_resu
lts"
[115] "unc.edu.b08923df-9b70-48eb-bc2b-023d1e1b0eeb.1322670.rsem.genes.normalized_resu
lts"
[116] "unc.edu.22366c3f-bf63-421e-b086-0ffb87945bf9.1327477.rsem.genes.normalized_resu
lts"
[117] "unc.edu.cfa04d49-cec6-4454-b10e-8b4600485f12.1326042.rsem.genes.normalized_resu
lts"
[118] "unc.edu.96b3c91a-389e-48cc-9347-ed65fa70f3de.1326718.rsem.genes.normalized_resu
lts"
[119] "unc.edu.dbeac9fe-cb6a-464c-9a96-d586797b1da0.1324935.rsem.genes.normalized_resu
lts"
[120] "unc.edu.f835bdde-2b5e-4013-a8d4-0074f66c9317.1573327.rsem.genes.normalized_resu
lts"
[121] "unc.edu.aca30642-6479-4cfb-bcac-b9b5e45c5686.1572835.rsem.genes.normalized_resu
lts"
[122] "unc.edu.db0d3ef1-a8d7-4a5e-863e-f9e2717dee54.1943193.rsem.genes.normalized_resu
lts"
[123] "unc.edu.db1370cf-7ca5-45ed-bba6-751f28e39825.1911336.rsem.genes.normalized_resu
lts"
[124] "unc.edu.758f95a1-8aca-43d0-aff2-39566606f88d.1911718.rsem.genes.normalized_resu
```

```
lts"
[125] "unc.edu.3088dac7-68b6-42f3-82eb-ff2bd451d7c5.1905950.rsem.genes.normalized_resu
lts"
[126] "unc.edu.db0a4e20-b5ad-4034-ae70-1e3747cdaa1f.1984484.rsem.genes.normalized_resu
lts"
[127] "unc.edu.bdaceebb-1720-4ed0-b4e2-e3dec1d2335b.1905879.rsem.genes.normalized_resu
lts"
[128] "unc.edu.80c995f9-48bf-49a7-82ac-27d646b93e81.1905838.rsem.genes.normalized_resu
lts"
[129] "unc.edu.78dd0304-d459-4d26-b0ba-39a8078c59d0.1906088.rsem.genes.normalized_resu
lts"
[130] "unc.edu.9d7459be-af93-4bf3-8d54-df569a16ee12.1906105.rsem.genes.normalized_resu
lts"
[131] "unc.edu.777c61bc-aa43-4661-a1d3-7a2f07dc055a.1905859.rsem.genes.normalized_resu
lts"
[132] "unc.edu.7eb83c83-31e0-435b-8866-9d2764098800.1924143.rsem.genes.normalized_resu
lts"
[133] "unc.edu.439804ec-6110-4628-b3ea-fa807c346846.2078314.rsem.genes.normalized_resu
lts"
[134] "unc.edu.6499ffb8-c649-4881-8ca1-95d84842083b.1991517.rsem.genes.normalized_resu
lts"
[135] "unc.edu.768f6ac0-d742-4dda-a695-c75070a5511a.2078332.rsem.genes.normalized_resu
lts"
[136] "unc.edu.ca04916e-5edc-4481-81d0-b0cc56925005.2078279.rsem.genes.normalized_resu
lts"
[137] "unc.edu.bf1fd845-aa87-49e7-8bd9-1443d6a3799c.1965591.rsem.genes.normalized_resu
lts"
[138] "unc.edu.1dd50bb6-84d4-4e07-ba01-802f470b46dd.1965230.rsem.genes.normalized_resu
lts"
[139] "unc.edu.9713f322-bae0-47aa-88d5-e2c50a7bd687.1942565.rsem.genes.normalized_resu
lts"
[140] "unc.edu.b1235878-9ab0-4335-befc-869ffa79cfab.1942140.rsem.genes.normalized_resu
lts"
[141] "unc.edu.21e0230a-8314-4d80-afca-9326c34d1299.1984499.rsem.genes.normalized_resu
lts"
[142] "unc.edu.a5105fe6-6ba9-4977-a976-49d9a1b6f164.1965269.rsem.genes.normalized_resu
lts"
[143] "unc.edu.fae54004-1d4e-414e-a85e-667fc2cb89b5.1965306.rsem.genes.normalized_resu
lts"
[144] "unc.edu.83ca9e67-060b-45ec-9ea9-076a64ae8062.1965064.rsem.genes.normalized_resu
lts"
[145] "unc.edu.085abecb-d625-44e2-b699-34f9863a9b28.1965078.rsem.genes.normalized_resu
lts"
[146] "unc.edu.d0979d89-4efa-4e30-8510-8aa96a7c2f3d.2078179.rsem.genes.normalized_resu
lts"
[147] "unc.edu.17a290d7-8577-4d11-8842-86c2f48e1cac.1965025.rsem.genes.normalized_resu
lts"
[148] "unc.edu.6447a7cf-ae7a-4896-b00f-10c357eb8c8b.1111841.rsem.genes.normalized_resu
lts"
[149] "unc.edu.900735b7-3870-421e-8c87-3f4e86fc8dae.1205081.rsem.genes.normalized_resu
lts"
[150] "unc.edu.37f38cd6-6b65-4646-8ee1-d86ccc02fa75.1110593.rsem.genes.normalized_resu
lts"
[151] "unc.edu.7c6ef5e6-8116-4a21-ae62-88a5e420bcc1.1441195.rsem.genes.normalized_resu
lts"
[152] "unc.edu.4f0fbd7c-4768-4bf2-b6f4-372f28b1953f.1438467.rsem.genes.normalized_resu
lts"
[153] "unc.edu.6cf7d085-d60c-4456-9266-5031628f687c.1113914.rsem.genes.normalized_resu
lts"
[154] "unc.edu.b549484e-cce0-4f6f-994c-910b458de4de.1110240.rsem.genes.normalized_resu
lts"
[155] "unc.edu.69180b6c-8fef-4453-812d-212730a35ea7.1104645.rsem.genes.normalized_resu
lts"
[156] "unc.edu.a1022e37-586a-4c6a-a754-01709bad7a43.1104963.rsem.genes.normalized_resu
```

lts"
[157] "unc.edu.877fb28c-fcdd-4e75-8057-029c9ff31d26.1081181.rsem.genes.normalized_resu
lts"
[158] "unc.edu.a660dca3-d4cd-4d7e-93fd-6be7a622cc59.1079711.rsem.genes.normalized_resu
lts"
[159] "unc.edu.a237a685-c274-4cc9-ae4d-64398d2ba92e.1384661.rsem.genes.normalized_resu
lts"
[160] "unc.edu.c2972973-dec9-4723-8fc6-af9db22e1cde.1384179.rsem.genes.normalized_resu
lts"
[161] "unc.edu.16b48e8d-f339-45b2-8182-fe27212bc8da.1384723.rsem.genes.normalized_resu
lts"
[162] "unc.edu.ea0be0ea-2959-435e-a1c6-c5a3b4c52f51.1573211.rsem.genes.normalized_resu
lts"
[163] "unc.edu.0a8dbb5b-77b8-4eda-a67c-8cf842c12d53.2101714.rsem.genes.normalized_resu
lts"
[164] "unc.edu.2fe33b61-8a00-424b-b881-e2808b099022.1905625.rsem.genes.normalized_resu
lts"
[165] "unc.edu.f66bb574-487a-426d-9b17-799718300e1e.1507986.rsem.genes.normalized_resu
lts"
[166] "unc.edu.297fecfd-e9b8-4882-8a55-312f2a04c3f6.1221327.rsem.genes.normalized_resu
lts"
[167] "unc.edu.1671e6a6-1c3a-4ec7-b6b8-bd88f5ffcc60.1119817.rsem.genes.normalized_resu
lts"
[168] "unc.edu.c54cd39c-c7fc-4a0c-9d33-9c1f78c80871.1112230.rsem.genes.normalized_resu
lts"
[169] "unc.edu.0d423ac2-0d64-4956-a897-d7bc6e1293c2.1382295.rsem.genes.normalized_resu
lts"
[170] "unc.edu.dbe34afe-ea16-4faa-adab-9106a92dfde6.1228083.rsem.genes.normalized_resu
lts"
[171] "unc.edu.aa7b5f63-ac8e-45bf-80e9-be4a576426a5.1108161.rsem.genes.normalized_resu
lts"
[172] "unc.edu.dea6072e-021d-4984-87ba-2c624e07fa18.1507621.rsem.genes.normalized_resu
lts"
[173] "unc.edu.4b57bac2-6b38-435f-b935-8eb6a3369d1c.1228040.rsem.genes.normalized_resu
lts"
[174] "unc.edu.9a28482f-36a7-490f-9d22-a69f82fbfc05.1227837.rsem.genes.normalized_resu
lts"
[175] "unc.edu.f398c31b-c947-4ba7-84c0-6d3b36daaf3f.1220860.rsem.genes.normalized_resu
lts"
[176] "unc.edu.274a99f3-1b61-48f3-afab-11088c63f488.1208449.rsem.genes.normalized_resu
lts"
[177] "unc.edu.29f0cffb-22a7-40a7-964d-9b84d7c5b442.1208493.rsem.genes.normalized_resu
lts"
[178] "unc.edu.6a45204a-96c6-4617-a4cc-27f71c05170b.1094760.rsem.genes.normalized_resu
lts"
[179] "unc.edu.8c957960-d5a3-4d07-a3aa-6c13b16fff0e.1102325.rsem.genes.normalized_resu
lts"
[180] "unc.edu.02e43817-47d3-4436-bcfa-37dd510eec4c.1094660.rsem.genes.normalized_resu
lts"
[181] "unc.edu.fb59e5ad-650a-4620-af05-9e8cb6c402ea.1206655.rsem.genes.normalized_resu
lts"
[182] "unc.edu.dbe8b05d-9b51-4d93-9e04-9609a5dd4cb8.1098710.rsem.genes.normalized_resu
lts"
[183] "unc.edu.ddb17f24-f233-40dc-bbbd-731e50d4fce4.1093717.rsem.genes.normalized_resu
lts"
[184] "unc.edu.aa29c7b0-8641-4a59-8b17-c007b3cb8726.1092742.rsem.genes.normalized_resu
lts"
[185] "unc.edu.2cbd77bd-dece-4177-ace2-98d9d545b575.1229563.rsem.genes.normalized_resu
lts"
[186] "unc.edu.9334a61d-eb4f-4504-9f4d-b9d55a8733de.1229724.rsem.genes.normalized_resu
lts"
[187] "unc.edu.193873d4-f0ad-4e8d-918b-9beb83965483.1229286.rsem.genes.normalized_resu
lts"
[188] "unc.edu.ff74b4c6-e938-4a40-ab1a-84525349a62e.1229487.rsem.genes.normalized_resu

```
lts"
[189] "unc.edu.5cb476d8-eae8-453c-8157-fdb71b4d5efa.1905981.rsem.genes.normalized_resu
lts"
[190] "unc.edu.664ffa40-4508-40ae-9505-84a10136b362.1905975.rsem.genes.normalized_resu
lts"
[191] "unc.edu.4c893780-d86b-4b01-a423-ca099e4fc007.1900409.rsem.genes.normalized_resu
lts"
[192] "unc.edu.772f9a82-9a03-4bf0-b7ce-877b15e3d017.1572844.rsem.genes.normalized_resu
lts"
[193] "unc.edu.9214caa3-2ad2-41ff-ab77-cf22c4ccce97.1508159.rsem.genes.normalized_resu
lts"
[194] "unc.edu.7524ebc8-c57e-44d5-9a4e-b5ccce93bf55.1078801.rsem.genes.normalized_resu
lts"
[195] "unc.edu.597e959c-bab8-4234-b57b-1b8413de7955.1214218.rsem.genes.normalized_resu
lts"
[196] "unc.edu.90865a55-659d-409f-a865-bae9e46c4a7c.1370959.rsem.genes.normalized_resu
lts"
[197] "unc.edu.f84c748d-1296-44aa-b7b1-0795939a88fe.1384143.rsem.genes.normalized_resu
lts"
[198] "unc.edu.b0b8fd4b-d725-417b-b17b-4cb6a7136459.1326527.rsem.genes.normalized_resu
lts"
[199] "unc.edu.8684c13a-9445-45a8-b37f-ce2b04b218bf.1326278.rsem.genes.normalized_resu
lts"
[200] "unc.edu.97c75ab2-4b91-4c26-992e-ed1252805aac.1327303.rsem.genes.normalized_resu
lts"
[201] "unc.edu.13549fc9-6145-4853-88a6-39c78cb3ab0a.1326935.rsem.genes.normalized_resu
lts"
[202] "unc.edu.fa3d0703-ce5e-49a9-96a6-0777f8e81cd3.1326766.rsem.genes.normalized_resu
lts"
[203] "unc.edu.87e51ccb-9ded-43c6-bb1a-a8a85d2d9fa6.1572896.rsem.genes.normalized_resu
lts"
[204] "unc.edu.974f4223-c166-4ea2-a061-25e3f4b415e7.1570288.rsem.genes.normalized_resu
lts"
[205] "unc.edu.6addf668-7b4d-4e3d-9086-b95818648be2.1976623.rsem.genes.normalized_resu
lts"
[206] "unc.edu.5642f9d8-a84b-4ab6-b841-910ac4010e16.1900269.rsem.genes.normalized_resu
lts"
[207] "unc.edu.764bce37-06d7-41e7-83d9-ce803dabd5f0.1899138.rsem.genes.normalized_resu
lts"
[208] "unc.edu.ab39e99f-8967-4068-b1e2-49e1fe2298ff.1899297.rsem.genes.normalized_resu
lts"
[209] "unc.edu.ae82fe73-dc4c-403b-af98-1d01ad87e732.1965510.rsem.genes.normalized_resu
lts"
[210] "unc.edu.b30fef8e-9736-4f6d-9547-cfd9163ebbb9.1991337.rsem.genes.normalized_resu
lts"
[211] "unc.edu.3a401a11-c977-45b6-9900-87c6adb7dc09.1991419.rsem.genes.normalized_resu
lts"
[212] "unc.edu.b4e31dbe-0923-401e-a664-e779914254b8.1108618.rsem.genes.normalized_resu
lts"
[213] "unc.edu.860fa33f-75d9-480d-92e7-2a5a45b35433.1095347.rsem.genes.normalized_resu
lts"
[214] "unc.edu.8ccc7a37-a9a4-4d77-9126-c8513c20bb29.1103629.rsem.genes.normalized_resu
lts"
[215] "unc.edu.64f425a9-2260-4cd3-8ddd-c43ac410b1a4.1092657.rsem.genes.normalized_resu
lts"
[216] "unc.edu.0c96bb0f-11f1-421c-b2c5-55458ba95d97.1501965.rsem.genes.normalized_resu
lts"
[217] "unc.edu.0cfbefba-24ff-474b-8bfa-b44a1f71b48c.1204527.rsem.genes.normalized_resu
lts"
[218] "unc.edu.439820e6-1011-461e-8002-5af1470a7827.2078249.rsem.genes.normalized_resu
lts"
[219] "unc.edu.bf359688-8652-4266-b4fd-e2f8f2782a97.1588689.rsem.genes.normalized_resu
lts"
[220] "unc.edu.b31f6ff5-bd06-43d7-966e-426b787222e8.1991418.rsem.genes.normalized_resu
```

```
lts"
[221] "unc.edu.993626ac-469c-4d9d-8278-63fda45598c0.1990942.rsem.genes.normalized_resu
lts"
[222] "unc.edu.51705e9c-8423-4eb8-8839-09206d4d115b.1991110.rsem.genes.normalized_resu
lts"
[223] "unc.edu.a017b3e8-6e27-4f0c-aff2-166b73743991.1102403.rsem.genes.normalized_resu
lts"
[224] "unc.edu.8a8e816b-8067-4ffe-ac37-f3ac2e0a29c4.1094004.rsem.genes.normalized_resu
lts"
[225] "unc.edu.c2842301-c4ec-41c6-96f7-53701541d93c.1326991.rsem.genes.normalized_resu
lts"
[226] "unc.edu.84c8990e-7656-4c48-8e5a-b03e2013013d.1229600.rsem.genes.normalized_resu
lts"
[227] "unc.edu.f842dd09-8bea-47d4-8489-a9ac06ca5172.1373171.rsem.genes.normalized_resu
lts"
[228] "unc.edu.5893078d-d8cd-4c74-b0e2-d412e367710b.1368921.rsem.genes.normalized_resu
lts"
[229] "unc.edu.8deed508-4b81-4573-afc1-a3da5ca52c60.1327754.rsem.genes.normalized_resu
lts"
[230] "unc.edu.af100535-eaf2-49a6-b4bb-5a2b85a2d2c9.1976658.rsem.genes.normalized_resu
lts"
[231] "unc.edu.4af52410-54ab-4307-96a0-b80579d18e0b.1092446.rsem.genes.normalized_resu
lts"
[232] "unc.edu.db278ebc-bf83-43c0-8a60-a863786ea973.1208478.rsem.genes.normalized_resu
lts"
[233] "unc.edu.4b847934-8dcb-451a-a0f1-a5f59bbd33db.1081445.rsem.genes.normalized_resu
lts"
[234] "unc.edu.3163dcdb-7a0f-428d-94be-9fc140cca0ca.1079313.rsem.genes.normalized_resu
lts"
[235] "unc.edu.9b1118f0-743f-4186-b6cb-571ddb72bc74.1572972.rsem.genes.normalized_resu
lts"
[236] "unc.edu.708e83be-4726-4eb6-a72c-ad855942551c.1572697.rsem.genes.normalized_resu
lts"
[237] "unc.edu.1a331a89-0666-49c2-8f5c-915568d8cb31.1578235.rsem.genes.normalized_resu
lts"
[238] "unc.edu.c11b849f-04dd-4599-8073-e2431bd68dd1.1897579.rsem.genes.normalized_resu
lts"
[239] "unc.edu.7d313d92-13f0-49f2-bff6-20e4fdd09337.1991448.rsem.genes.normalized_resu
lts"
[240] "unc.edu.91128d24-c61a-47cf-938b-00ff80e4d0e7.1991314.rsem.genes.normalized_resu
lts"
[241] "unc.edu.38574f35-5845-4a7d-8657-da3d8757a0eb.1212842.rsem.genes.normalized_resu
lts"
[242] "unc.edu.36c6906f-7f3e-4bb4-9813-246dc7669fc6.1350967.rsem.genes.normalized_resu
lts"
[243] "unc.edu.e4ac001e-f87e-4c46-8e0e-cee8a4f80965.1352241.rsem.genes.normalized_resu
lts"
[244] "unc.edu.1fd32c7c-73dd-4c17-a0ac-7b33f610976e.1328777.rsem.genes.normalized_resu
lts"
[245] "unc.edu.2d6005de-b9ad-48ab-bfc9-60995838a002.1963302.rsem.genes.normalized_resu
lts"
[246] "unc.edu.00481f66-1aad-4885-9162-bf802b1ed7f5.2064374.rsem.genes.normalized_resu
lts"
[247] "unc.edu.a3b81644-c1b8-4d12-b6f8-6160533758cc.2077674.rsem.genes.normalized_resu
lts"
[248] "unc.edu.38843819-bf57-4c30-ae2f-408653860739.2065446.rsem.genes.normalized_resu
lts"
[249] "unc.edu.0d7719a9-58ac-4c27-99d3-0a6321c03ef6.2065234.rsem.genes.normalized_resu
lts"
[250] "unc.edu.6b636016-1b27-4977-8632-85ce773f7db9.2065901.rsem.genes.normalized_resu
lts"
[251] "unc.edu.27a878b3-d7eb-49e3-9e1f-a12fcc33d67d.2065161.rsem.genes.normalized_resu
lts"
[252] "unc.edu.fd1831a1-568b-4292-8d70-1450c392825f.2234133.rsem.genes.normalized_resu
```

lts"
[253] "unc.edu.da2c0992-74c0-4380-8c6c-c9ffa73767f6.2064392.rsem.genes.normalized_resu
lts"
[254] "unc.edu.38534c34-666f-4319-86b8-8243ab76cb40.2064317.rsem.genes.normalized_resu
lts"
[255] "unc.edu.f2510b83-536a-4d29-bca7-ff3ca3ca9e44.2063980.rsem.genes.normalized_resu
lts"
[256] "unc.edu.6028a290-48e8-400f-9ab6-39beda6051cd.2115753.rsem.genes.normalized_resu
lts"
[257] "unc.edu.a117b181-a01e-49a7-a82b-13f4a385dd45.2013436.rsem.genes.normalized_resu
lts"
[258] "unc.edu.704d2001-9501-4426-b751-af5fc74a6378.2013850.rsem.genes.normalized_resu
lts"
[259] "unc.edu.dc1f01da-cff9-4b61-b743-0452e023e2a1.2097532.rsem.genes.normalized_resu
lts"
[260] "unc.edu.ca467949-9170-4c3f-8e67-5b7762d31d4a.2108365.rsem.genes.normalized_resu
lts"

DATASET4:
Mutation Calling Files:
hgsc.bcm.edu_SKCM.IlluminaGA_DNASeq.1.somatic.maf
PR_TCGA_SKCM_PAIR_Capture_All_Pairs_QCPASS_v4.aggregated.capture.tcga.uuid.automated.s
omatic.maf

TCGA_SKCM RNASeq downloaded from TCGA portal Jan 28, 2015
[1] "unc.edu.c8600bba-b4bd-400e-ab67-03d66be9da31.1289104.rsem.genes.normalized_resul
ts"
[2] "unc.edu.2e00b580-b6b7-433e-88e8-69a75fae592f.1385064.rsem.genes.normalized_resul
ts"
[3] "unc.edu.194ed685-060c-4577-99b9-86238e24c17c.1218433.rsem.genes.normalized_resul
ts"
[4] "unc.edu.eea5dc11-94fe-4ebf-b352-f70c2d358236.1237643.rsem.genes.normalized_resul
ts"
[5] "unc.edu.a4a2d2e8-d2cd-4551-942d-8cc066d332a6.1248563.rsem.genes.normalized_resul
ts"
[6] "unc.edu.a43aa841-8bb9-467b-8e51-76c30d5856df.1286259.rsem.genes.normalized_resul
ts"
[7] "unc.edu.05e46b8a-14f5-4315-9418-25bc9de5c436.2066619.rsem.genes.normalized_resul
ts"
[8] "unc.edu.279e2d3b-f060-4afa-a26e-673a4af821d4.1971438.rsem.genes.normalized_resul
ts"
[9] "unc.edu.73a8105e-c356-4a7e-888c-feb5339074fb.1981805.rsem.genes.normalized_resul
ts"
[10] "unc.edu.1d1a1d5e-fdfe-4251-93ec-c89b65bb341e.1982307.rsem.genes.normalized_resul
ts"
[11] "unc.edu.4f8a1027-ed36-4171-8d23-c6a4b089fe5e.1982378.rsem.genes.normalized_resul
ts"
[12] "unc.edu.3b2af3d9-31f1-4075-8a42-2e4f371cd0a6.2147749.rsem.genes.normalized_resul
ts"
[13] "unc.edu.6cf6c8d5-4883-40a2-9a5c-0041f8f073de.2147625.rsem.genes.normalized_resul
ts"
[14] "unc.edu.11e4b666-d6e8-4b8d-9340-5ac8ac3979e6.1286721.rsem.genes.normalized_resul
ts"
[15] "unc.edu.5d4390cc-c091-4844-b40c-f936ab881e61.1251031.rsem.genes.normalized_resul
ts"
[16] "unc.edu.641b06e6-a787-4ad1-9bfa-ea27eb675829.1251093.rsem.genes.normalized_resul
ts"
[17] "unc.edu.e1be8377-176c-4f4c-b3aa-86bbb6cd0f3a.1239854.rsem.genes.normalized_resul
ts"
[18] "unc.edu.8b4c42a9-7d09-4989-a0e2-43abb6097137.1642553.rsem.genes.normalized_resul
ts"
[19] "unc.edu.57e2418c-e87f-4dcd-9e9d-1e2eb4e68ef0.1846713.rsem.genes.normalized_resul
ts"
[20] "unc.edu.d19e0e12-91bc-4ba0-b966-f29e3ba9c1c4.1971704.rsem.genes.normalized_resul

```
ts"
[21] "unc.edu.83b592f7-21ce-4e32-aaf4-3f9402f48284.1982576.rsem.genes.normalized_resul
ts"
[22] "unc.edu.eca521e3-f15c-4709-9a2d-ce6c34dcf342.1286927.rsem.genes.normalized_resul
ts"
[23] "unc.edu.24e9d763-4fc9-4806-90f7-6aeda279bc8f.1092672.rsem.genes.normalized_resul
ts"
[24] "unc.edu.8fba87be-9062-49e8-ab5d-95b4c4c6a721.1240919.rsem.genes.normalized_resul
ts"
[25] "unc.edu.0fce8c31-15af-4558-9c50-341d447ff5d5.1228667.rsem.genes.normalized_resul
ts"
[26] "unc.edu.58d8d333-2db3-414a-b8c6-22e28d79403b.1226600.rsem.genes.normalized_resul
ts"
[27] "unc.edu.cc38cfd7-4ade-4834-812d-109d152a45b8.1247400.rsem.genes.normalized_resul
ts"
[28] "unc.edu.5ac1f533-a9f3-4a5e-a25f-4b04ffcf50b6.1041973.rsem.genes.normalized_resul
ts"
[29] "unc.edu.c0e841a4-6638-4e17-a3eb-5a900f616b0c.1240918.rsem.genes.normalized_resul
ts"
[30] "unc.edu.34049292-a4b9-4a3a-94be-e1febcecc848.1225076.rsem.genes.normalized_resul
ts"
[31] "unc.edu.32cf555c-9de8-4de4-ba4a-a729ac88e8ba.1223398.rsem.genes.normalized_resul
ts"
[32] "unc.edu.0d53cf3e-63d6-465b-b1b6-763b9a29dc64.1287994.rsem.genes.normalized_resul
ts"
[33] "unc.edu.89d28931-1552-4274-8abb-9fba776c06f7.1212493.rsem.genes.normalized_resul
ts"
[34] "unc.edu.6e4145d0-e417-4690-8956-c5d1c1194fe3.1221971.rsem.genes.normalized_resul
ts"
[35] "unc.edu.ade35c8d-30dd-4e45-9f7c-9818072a02db.1286366.rsem.genes.normalized_resul
ts"
[36] "unc.edu.1fd474c9-0ccd-44f3-bf7b-9bb6a2a1e297.1220350.rsem.genes.normalized_resul
ts"
[37] "unc.edu.b8a63774-db74-4cd6-9ac0-065f1126448d.1224701.rsem.genes.normalized_resul
ts"
[38] "unc.edu.08997d9f-db88-4f45-9d76-772cb9acc75d.1220790.rsem.genes.normalized_resul
ts"
[39] "unc.edu.55ad742e-cc71-4ebe-9f91-8242d60b46e0.1221596.rsem.genes.normalized_resul
ts"
[40] "unc.edu.faa7c094-e5c2-4ae7-b984-7377fcfff9ac.1221644.rsem.genes.normalized_resul
ts"
[41] "unc.edu.315e9fee-404c-4f59-829b-d769e965c87e.1385479.rsem.genes.normalized_resul
ts"
[42] "unc.edu.1dcb8c9f-677e-4884-a119-50460646ba33.1386643.rsem.genes.normalized_resul
ts"
[43] "unc.edu.cb033feb-06ad-4674-bc0b-6539efb9aa85.1319573.rsem.genes.normalized_resul
ts"
[44] "unc.edu.a4c3676d-fc5d-4d26-8627-6f494657b84a.1385133.rsem.genes.normalized_resul
ts"
[45] "unc.edu.3ada0d86-a7dd-4ef8-ab88-bff478010c5e.1319653.rsem.genes.normalized_resul
ts"
[46] "unc.edu.dca3aac8-2cc3-41fb-b030-0b0084ca6217.1387561.rsem.genes.normalized_resul
ts"
[47] "unc.edu.862b334e-5cdd-4e05-b2db-8a5cbe64b097.1227016.rsem.genes.normalized_resul
ts"
[48] "unc.edu.7c23f39f-6b3f-41d8-bb17-c35788b2b060.1227148.rsem.genes.normalized_resul
ts"
[49] "unc.edu.f3320aca-c30a-4542-9778-2fd278d9bb83.2095015.rsem.genes.normalized_resul
ts"
[50] "unc.edu.a6d27c0b-24c5-4633-a6d1-3f7f6c8525fd.1248731.rsem.genes.normalized_resul
ts"
[51] "unc.edu.ce894d3c-c781-4e3a-a32c-1b3f85c84df3.1246643.rsem.genes.normalized_resul
ts"
[52] "unc.edu.a44ecade-befb-4514-9873-03c70438d9c7.1860702.rsem.genes.normalized_resul
```

ts"
[53] "unc.edu.2a382958-2b06-4f44-8e21-e2a5bf2f5d15.1563331.rsem.genes.normalized_resul
ts"
[54] "unc.edu.261b4ef8-1e68-4179-826b-8c4122502df4.2139054.rsem.genes.normalized_resul
ts"
[55] "unc.edu.454aa611-1004-4e70-ba50-276f75b72113.2139049.rsem.genes.normalized_resul
ts"
[56] "unc.edu.400b226d-d5a5-444a-9b0e-33c6c7252d1e.1213230.rsem.genes.normalized_resul
ts"
[57] "unc.edu.c55d0f65-fa13-4bb5-a556-2bd78bac2ccf.1228105.rsem.genes.normalized_resul
ts"
[58] "unc.edu.7cecef67-73bf-4003-821c-e42ce6adfab7.2250211.rsem.genes.normalized_resul
ts"
[59] "unc.edu.4eadf1bf-2a42-4de6-9710-86350c2fe144.2246596.rsem.genes.normalized_resul
ts"
